# AURKA destruction is decoupled from its activity at mitotic exit but is essential to suppress interphase activity

**DOI:** 10.1242/jcs.243071

**Published:** 2020-06-16

**Authors:** Ahmed Abdelbaki, H. Begum Akman, Marion Poteau, Rhys Grant, Olivier Gavet, Giulia Guarguaglini, Catherine Lindon

**Affiliations:** 1 Department of Pharmacology, University of Cambridge, Tennis Court Road, Cambridge CB2 1PD, UK; 2 Institut Gustave Roussy, UMR9019 – CNRS, 114 rue Edouard Vaillant, 94805 Villejuif, France; 3 Institute of Molecular Biology and Pathology, CNR, Via degli Apuli 4, 00185 Roma, Italy

**Keywords:** Aurora A kinase, Cdh1, FZR1, TPX2, Mitochondria, Mitosis

## Abstract

Activity of AURKA is controlled through multiple mechanisms including phosphorylation, ubiquitin-mediated degradation and allosteric interaction with TPX2. Activity peaks at mitosis, before AURKA is degraded during and after mitotic exit in a process strictly dependent on the APC/C coactivator FZR1. We used FZR1 knockout cells (FZR1^KO^) and a novel FRET-based AURKA biosensor to investigate how AURKA activity is regulated in the absence of destruction. We found that AURKA activity in FZR1^KO^ cells dropped at mitotic exit as rapidly as in parental cells, despite absence of AURKA destruction. Unexpectedly, TPX2 was degraded normally in FZR1^KO^ cells. Overexpression of an N-terminal TPX2 fragment sufficient for AURKA binding, but that is not degraded at mitotic exit, caused delay in AURKA inactivation. We conclude that inactivation of AURKA at mitotic exit is determined not by AURKA degradation but by degradation of TPX2 and therefore is dependent on CDC20 rather than FZR1. The biosensor revealed that FZR1 instead suppresses AURKA activity in interphase and is critically required for assembly of the interphase mitochondrial network after mitosis.

This article has an associated First Person interview with the first authors of the paper.

## Abstract (precis)

**Summary:** Destruction of AURKA plays no role in inactivating the kinase at mitotic exit but is required to keep AURKA activity low in interphase so that a mitochondrial network can be reassembled.

## INTRODUCTION

Aurora kinase A (AURKA) is a major mitotic kinase required for multiple steps in mitosis, including centrosome maturation, microtubule nucleation and organization into a bipolar spindle and mitotic checkpoint function (Barr and Gergely, 2007; Courtheoux et al., 2018). Recent studies of the effects of acute inhibition of AURKA during mitosis have concluded that it plays an important role during mitotic exit in regulating assembly of the anaphase spindle upon which sister chromatids are segregated (Hégarat et al., 2011; Lioutas and Vernos, 2013; Reboutier et al., 2013). AURKA also has a number of non-mitotic roles that include disassembly of the primary cilium, regulation of myc family transcription factors and response to replication stress (Büchel et al., 2017; Byrum et al., 2019; Cantor, 2019; Otto et al., 2009; Plotnikova et al., 2012). We and others recently reported that AURKA constitutively regulates mitochondrial morphology and function (Bertolin et al., 2018; Grant et al., 2018) in addition to a previously reported role in promoting mitochondrial fission in mitosis (Kashatus et al., 2011). Given the central role of mitochondrial homeostasis in cell fate and function, this newly identified role for AURKA lends importance to the question of when AURKA is present in the cell cycle, and whether it is active.

Widespread reports in the literature of interphase activity of AURKA, and of its activation through a number of interacting partners (Nikonova et al., 2013), can be explained by the unique allosteric properties of this kinase. Either autophosphorylation of the T-loop (Bayliss et al., 2003; Eyers et al., 2003; Tsai et al., 2003) or allosteric interactors (Dodson and Bayliss, 2012; Levinson, 2018; Ruff et al., 2018; Zorba et al., 2014) contribute to the active conformation of AURKA. The most prominent of these allosteric activators is TPX2, which controls AURKA localization, activation and stability on the mitotic spindle during mitosis (Bayliss et al., 2003; Giubettini et al., 2011; Kufer et al., 2002). Interaction with TPX2, whilst protecting the AURKA autophosphorylated site (pT288 in hsAURKA) from access by PP1 phosphatase (Bayliss et al., 2003), also acts to stabilize the T-loop with or without its phosphorylation. Consequently, phosphatase-mediated reversal of T288 phosphorylation may not be sufficient to eliminate activity of AURKA.

In somatic cells, both AURKA protein and kinase activity are low or undetectable through much of the cell cycle but rise sharply in G2 phase, most prominently on the duplicated centrosomes and then on the bipolar spindle in mitosis. pT288 staining is almost entirely confined to the centrosomes, in line with the idea that autophosphorylation is just one route to activation of AURKA. Indeed, a conformational sensor of AURKA detects active kinase on spindle poles, as expected, but also in the cytoplasm, both during and after mitosis (Bertolin et al., 2016). Mitotic destruction of AURKA begins late in anaphase following assembly of the spindle midzone and at the time of spindle pole disassembly, in a manner that depends on the Cdh1 co-activator (hereafter referred to as FZR1) of the anaphase promoting complex/cyclosome (APC/C) (Castro et al., 2002; Floyd et al., 2008; Littlepage and Ruderman, 2002; Taguchi et al., 2002).

The APC/C is responsible for the destruction of dozens of substrates during mitotic exit and coordinates mitotic exit with the processes of chromosome segregation and cytokinesis through the concerted regulation of its coactivators (Pines, 2011). APC/C–CDC20 initially targets cyclin B and securin during metaphase to drive chromosome segregation and mitotic exit. After anaphase onset, altered substrate specificity of APC/C–CDC20 and activation of APC/C–FZR1 together control degradation of the remaining pool of cyclin B (Afonso et al., 2019) as well as other APC/C substrates including Aurora kinases (Lindon et al., 2015). Unlike most substrates, AURKA and AURKB show strict dependence on FZR1 for their destruction in cell-based assays (Clijsters et al., 2013; Floyd et al., 2008; Min et al., 2015). Neither the molecular basis, nor the functional significance of this specificity in Aurora kinase targeting, is understood.

Given the complexity of AURKA regulation during the cell cycle, we investigated the contribution of AURKA destruction to its inactivation during mitotic exit using novel tools in the form of a CRISPR/Cas9 FZR1 knockout cell line and a FRET-based AURKA activity biosensor that we have recently generated. We conclude from our studies that FZR1-mediated destruction of AURKA plays no role in the timing of its inactivation during mitotic exit but is critical to suppress interphase activity and function of the kinase. We test this idea by demonstrating that re-establishment of mitochondrial connectivity after mitosis requires suppression of activity of undegraded AURKA.

## RESULTS

**Fig. 1.**
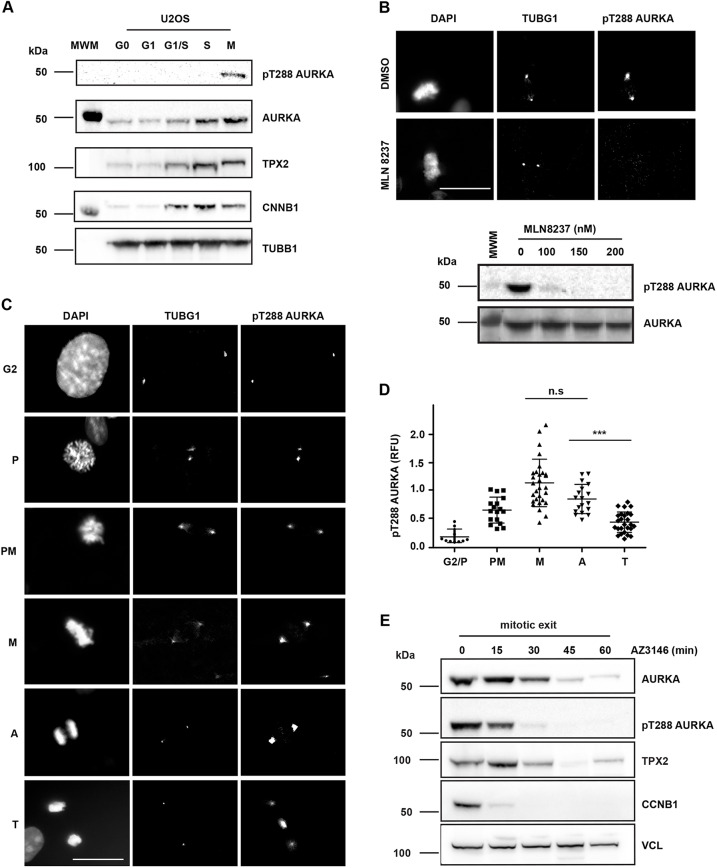
**Inactivation of AURKA during mitotic exit begins at anaphase.** (A) pT288 antibody detects active phosphorylated AURKA only in mitotic cells. U2OS cells were synchronized as described in the Materials and Methods and blotted for pT288, total AURKA and other mitotic markers (TPX2, CNNB1 and TUBB1). MWM indicates molecular weight marker lane. (B) pT288 signal is sensitive to the AURKA-specific inhibitor MLN8237, as shown by IF of mitotic cells from a MeOH-fixed unsynchronized population (upper panel) or by immunoblot of STLC-arrested mitotic cells treated for 3 h at the indicated doses (lower panel). AURKA-specific pT288 signal is restricted to centrosomes and spindle pole bodies (marked by γ-tubulin, TUBG1). DNA is shown by DAPI staining. DMSO indicates images of vehicle-treated control cells. MWM indicates molecular weight marker lane. See also Fig. S1. (C–E) Quantification of pT288-AURKA during mitotic exit. In C and D, unsynchronized cell populations were fixed and stained as in B. Cells were judged to be at different stages of mitosis according to DAPI staining (C) and scored for mean pT288 AURKA signal measured in a fixed region of interest centered on TUBG1 signal at centrosomes or spindle poles (D). Scatter plots show distribution and mean±s.d. of pooled data from two independent experiments. Data are normalized to the mean value at metaphase. The plot is representative of two biological replicates. G2 and prophase (P), *n*=10; prometaphase (PM), *n*=15; metaphase (M), *n*=30; anaphase (A), *n*=30 and telophase (T), *n*=26. M versus A, not significant (n.s.); A versus T, *P*<0.0001 (***); Student's *t*-test. RFU, relative fluorescence units. For the western blots shown in E, cells were synchronized in 5 μM STLC and released by checkpoint inhibition using 10 μM AZ3146, with extracts harvested at the times indicated. These were examined by immunoblotting for AURKA, pT288-AURKA and TPX2 levels. Disappearance of cyclin B1 (CCNB1) acts as marker for mitotic exit, and the level of vinculin (VCL) acts as loading control. Data shown in A is from a single experiment, data shown in B and E are representative of two experiments. Scale bars: 10 μm.

Although AURKA activity has been described to peak in M phase there has been no detailed characterization of how AURKA activity varies throughout mitosis. In particular the question of how AURKA activity is regulated during mitotic exit, given that AURKA also has anaphase functions (Afonso et al., 2017; Reboutier et al., 2015), and the importance of its ongoing destruction for attenuation of activity, remain unresolved. We first used a commercially available antibody against the activated T-loop of Aurora kinases (pT288 in AURKA) that confirmed by immunoblot the peak of active AURKA at mitosis in extracts from synchronized U2OS cells (Fig. 1A). We then used the same antibody to stain fixed mitotic cells for immunofluorescence (IF), marking centrosomes with γ-tubulin co-stain. All phospho-epitope signal on the centrosomes and spindle poles was abolished by treatment with the AURKA inhibitor MLN8237, whereas the signal at the midbody (where the same antibody recognizes pT232 of AURKB) was insensitive to 100 nM MLN8237, indicating that the centrosomal pT288 signal is specific to AURKA as expected (Fig. 1B; Fig. S1) (Asteriti et al., 2014; de Groot et al., 2015). We quantified fluorescence intensity associated with pT288 at different stages of mitosis, scored according to DNA morphology. We found that pT288 was evident at the centrosomes from G2 phase onwards. Following nuclear envelope breakdown (NEB), prometaphase (PM) cells showed a strong increase in centrosome- and spindle pole-associated pT288 signal, peaking at metaphase. pT288 signal began to decline in anaphase cells before dropping sharply in telophase cells (Fig. 1C,D). We concluded that inactivation of AURKA, measured as a decrease in pT288 signal, occurs at approximately the time of onset of AURKA destruction, which occurs around 10 min after anaphase onset in human cells (Floyd et al., 2008). We further analysed AURKA inactivation kinetics by immunoblot analysis of extracts from cells synchronized through mitotic exit. Cells were synchronized in mitosis using Eg5 (also known as KIF11) inhibitor STLC to trigger the spindle assembly checkpoint (SAC), then treated with Mps1 (also known as TTK) inhibitor (AZ3146) to override SAC-mediated mitotic arrest. Immunoblotting of cell extracts showed that under such conditions of ‘forced’ mitotic exit, cells significantly degraded cyclin B1 – the trigger for anaphase entry – within 15 min (Fig. 1E). The fall in pT288 and total AURKA levels were delayed relative to cyclin B1 degradation, consistent with events ongoing through anaphase and telophase into the following G1 phase.

**Fig. 2.**
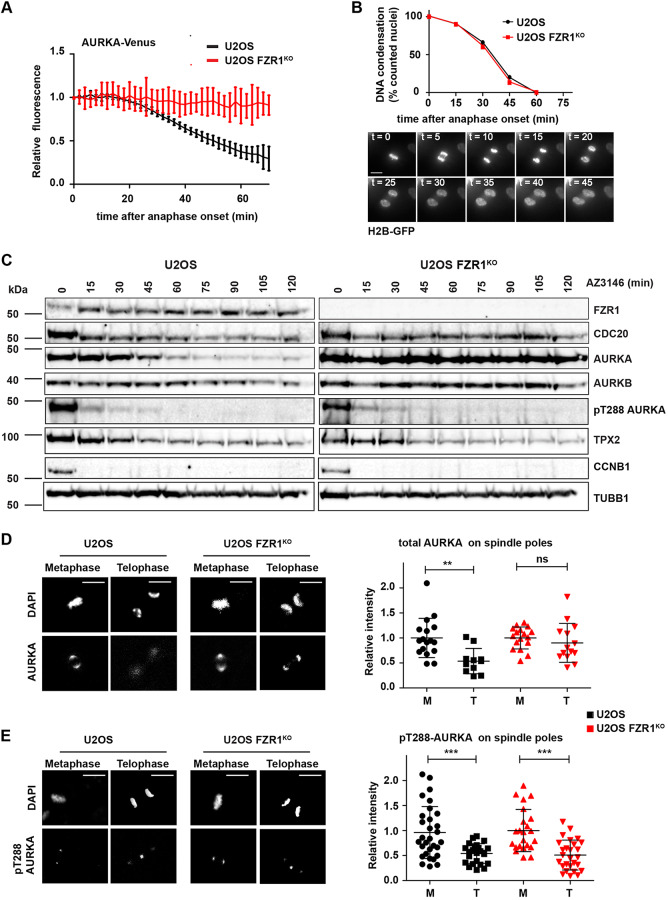
**AURKA destruction is not required for pT288-AURKA down-regulation at mitotic exit.** (A,B) There is no mitotic exit destruction of AURKA in FZR1 KO cells (FZR1^KO^). AURKA–Venus (A) or H2B–GFP (B) were transiently transfected into both U2OS and U2OS FZR1^KO^ cells. In A, quantifications of total fluorescence measurements from single mitotic cells were used to generate degradation curves for AURKA–Venus. Fluorescence values for individual curves were normalized to the last frame before anaphase onset, and all curves were *in silico* synchronized to anaphase. *n*=10 cells. Data are mean±s.d. In B, H2B–GFP fluorescence was used to score DNA as condensed or decondensed in cells undergoing mitotic exit (example shown in lower panels). Percentage of cells with condensed DNA over time was plotted as a measure of cumulative mitotic exit. *n*≥10 cells. (C–E) AURKA activity scored by pT288 is not affected by FZR1^KO^ during mitotic exit. For the western blots in C, U2OS and FZR1^KO^ cells were synchronized to prometaphase using 5 μM STLC and released by checkpoint inhibition using 10 μM AZ3146, with extracts harvested at the times indicated. Lysates were analysed by immunblot with antibodies against AURKA, pT288-AURKA and other mitotic regulators and are representative of three independent experiments. D and E show AURKA and pT288-AURKA staining associated with individual centrosomes/spindle poles in metaphase (M) versus telophase (T) cells (left-hand panels). Fluorescence values were measured as in Fig. 1 and are presented as scatter plots, with mean±s.d. indicated, for total AURKA (D) and pT288-AURKA (E) in both U2OS and FZR1^KO^. All values were normalized to the mean value from control metaphase cells. ns, not significant; **P<0.001; ***P<0.0001 (Student's *t*-test). D, *n*≥11 from one experiment; E, *n*≥23 from two experiments. Scale bars: 10 μm.

We investigated whether AURKA destruction contributes to the fall in kinase activity at mitotic exit. Mitotic AURKA destruction is critically dependent on the FZR1 co-activator of APC/C, so we used a FZR1 knockout (FZR1^KO^) in U2OS cells generated by CRISPR/Cas9 (Fig. S2) to study AURKA inactivation in the absence of its destruction at mitotic exit. Indeed, in FZR1^KO^ cells, AURKA–Venus protein levels measured in single-cell degradation assays remained constant after anaphase onset, compared to levels in the the parental U2OS cell line (Fig. 2A). We further determined that there was no delay in mitotic exit in FZR1^KO^ cells that could account for the stability of AURKA, measuring the elapsed time from anaphase onset to completed DNA decondensation during unperturbed mitotic exit in parental and FZR1^KO^ U2OS cells expressing H2B–GFP (Fig. 2B) or under conditions of forced mitotic exit (Fig. S2). We found that mitotic exit is in fact slightly accelerated in FZR1^KO^ cells, as previously reported using siRNA-mediated suppression of FZR1 (Floyd et al., 2008). We then compared loss of pT288 staining during mitotic exit between parental and FZR1^KO^ cells. We found by immunoblot that loss of pT288 signal was identical in both cell lines despite strong stabilization of the AURKA signal during mitotic exit in FZR1^KO^ cells (Fig. 2C; Fig. S2). AURKB was also stabilized but to a lesser extent, consistent with slower degradation of AURKB (Lindon et al., 2015). We note that degradation of endogenous TPX2 during mitotic exit, like inactivation of AURKA, was insensitive to FZR1 knockout, and appeared more complete in FZR1^KO^ cells, where CDC20 levels persisted compared to levels in parental cells (Fig. 2C). TPX2 was first proposed as a substrate of APC/C–FZR1 (Stewart and Fang, 2005) but our results suggest that, in an unperturbed mitotic exit, TPX2 – like cyclin B1 (CCNB1) – is a substrate for CDC20 before FZR1 activation. We then fixed parental U2OS and FZR1^KO^ cells and processed them for quantitative IF analysis of pT288-AURKA and total AURKA staining at spindle poles in metaphase and telophase. In this analysis AURKA persisted on spindle poles during mitotic exit in FZR1^KO^ cells compared to levels in parental U2OS (Fig. 2D). In both cell lines, active AURKA, as measured by T-loop phosphorylation of the kinase (pT288), was strongly reduced after mitotic exit, independent of the residual levels of protein (Fig. 2E). We confirmed that the persistence of AURKA on spindle poles is a direct effect of AURKA non-degradation by showing that wild-type AURKA–Venus, but not a non-degradable version, was rapidly lost from spindle poles in unperturbed mitotic exit (Fig. S3).

**Fig. 3.**
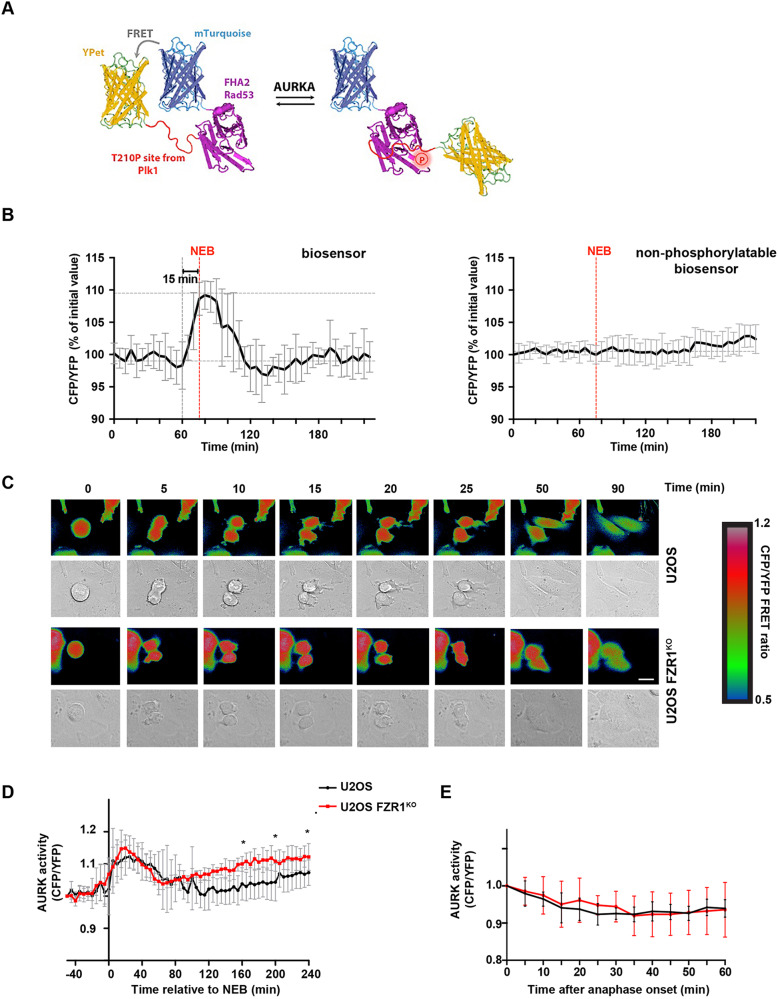
**A FRET-based biosensor records unaltered** **parameters of mitotic AURKA activation and inactivation in FZR1^KO^ cells.** (A) Schematic illustration of the AURKA biosensor showing high FRET (left) versus low FRET state (right). (B) Inverted FRET measurements (CFP/YFP emission) from timelapse movies of cells expressing the biosensor, or a non-phosphorylatable version, show that the biosensor reports on mitotic phosphorylation events, *n*≥8. Data are mean±s.d. Further characterization of the specificity of the biosensor is shown in Fig. S4. (C) Examples of inverted false-coloured FRET ratio of biosensor-expressing single U2OS and FZR1^KO^ cells passing through mitosis. High FRET (blue) reports on the non-phosphorylated state, whereas low FRET (red) reports on the phosphorylated probe. Scale bar: 10 μm. (D,E) FRET ratio values measured using the biosensor show that total Aurora kinase (AURK) activity is normally regulated through mitosis in FZR1^KO^ cells but rises again in G1 phase (*P<0.05, Student's *t*-test). In D, cells are *in silico* synchronized to NEB. In E, cells are *in silico* synchronized and FRET values normalized to anaphase onset. Data are mean±s.d. of *n*=8 cells.

A number of AURKA binding partners have been shown to affect the activity of AURKA, some acting independently of T-loop phosphorylation on T288, via distinct effects on its conformational dynamics. Therefore, we developed a diffusible kinase biosensor, based on the well-established design of a fluorescent protein FRET pair separated by a phospho-threonine binding domain and specific phosphorylation motif (Violin et al., 2003), to provide a cell-wide readout of AURKA activity in living single cells as they progress through mitosis. This AURKA-directed FRET biosensor was created using the T210 motif from the T-loop of PLK1, a well-established target of AURKA (Macůrek et al., 2008; Seki et al., 2008). Phosphorylation of the T210 motif in the biosensor caused loss of FRET in mitotic cells, measured as an increase in CFP/YFP emission ratio of ∼10%, in a manner dependent on its phosphorylation site and sensitive to specific inhibitors of AURKA (Fig. 3A,B; Fig. S4). Some sensitivity to inhibitors of AURKB at higher doses indicated that the biosensor might not be completely specific to AURKA (although selective AURKB inhibitors might also target AURKA at these concentrations) (Fig. S4). This finding was not unexpected for a diffusible biosensor, since part of the specificity in substrate phosphorylation by Aurora kinases is proposed to reside in the co-localization of the kinase with its substrates (de Groot et al., 2015; Hégarat et al., 2011). We then used this biosensor in U2OS and FZR1^KO^ cells to measure Aurora kinase activity at mitosis and following mitotic exit. FRET measurements in mitotic cells were in agreement with the analysis of pT288 staining (Fig. 2), in showing that 1/FRET signal started to increase in late G2, peaking after NEB and decaying during mitotic exit and into interphase. We found that the increase in AURKA activity measured at mitotic entry was identical in individual U2OS and FZR1^KO^ cells when the FRET measurements for each cell were synchronized to NEB *in silico*. *In silico* synchronization to anaphase onset revealed that the timing of Aurora kinase inactivation was identical in U2OS and FZR1^KO^ cells (Fig. 3C,D; Fig. S4), and when FRET signals were normalized to the anaphase onset value, the inactivation curves were directly superimposable (Fig. 3E). Therefore, mitotic Aurora kinase inactivation is independent of FZR1-dependent degradation. As shown in Fig. 3D, FRET measurements returned to pre-mitotic levels at about 1 h after anaphase onset, indicating complete inactivation of the mitotic pool of AURKA. We observed, however, that FZR1^KO^ cells built up AURKA activity after mitosis earlier than parental cells and reached a significantly higher level in G1 phase (Fig. 3D). We concluded that destruction of AURKA does not influence its inactivation during mitotic exit but might be important to prevent premature reactivation early in the cell cycle.

**Fig. 4.**
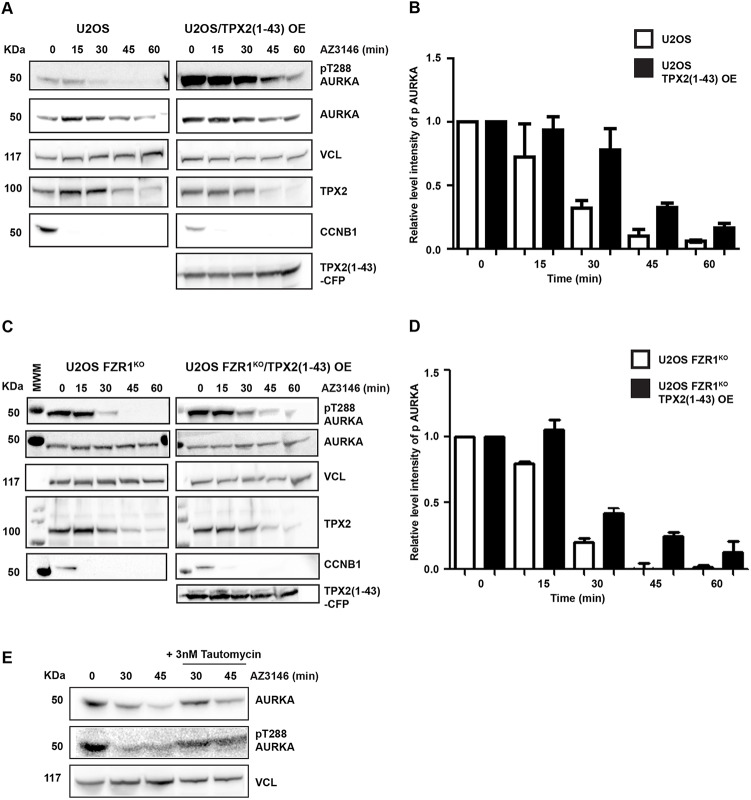
**AURKA inactivation is controlled through TPX2.** U2OS (A,B) and FZR1^KO^ (C,D) cells were transfected with TPX2(1–43)–CFP and synchronized through mitotic exit as described in the legend to Fig. 2. Quantitative immunoblotting of cell lysates shows that loss of pT288-AURKA during mitotic exit is delayed in the presence of TPX2(1–43) in both parental and FZR1^KO^ cells. Cyclin B1 (CCNB1) is used as a marker for mitotic exit, level of vinculin (VCL) is shown as a loading control. MWM indicates molecular weight marker lane. Bar charts (B,D) show pT288 signal normalized against vinculin. Results presented are mean±s.d. values from three independent experiments. (E) AURKA inactivation is phosphatase-dependent. U2OS cells undergoing mitotic exit were treated with 3 nM PP1 inhibitor tautomycin 10 min after relief of SAC arrest by AZ3146. Lysates harvested at the indicated time points after AZ3146 treatment were subject to immunoblot analysis. Blots shown are representative of two independent experiments.

How can we explain the timing of AURKA inactivation at mitotic exit? We have previously shown that interaction with TPX2 stabilizes AURKA against APC/C–FZR1-mediated degradation (Giubettini et al., 2011), and from this concluded that loss of interaction with TPX2 contributes to the timing of AURKA degradation at mitotic exit. Our finding here that AURKA degradation plays no role in its inactivation led us to evaluate the hypothesis that AURKA inactivation could in fact depend on TPX2 degradation. One way to test this hypothesis would be through expression of a non-degradable version of TPX2. Because transient overexpression of full-length TPX2 inhibited mitotic progression in our hands, we used an N-terminal fragment of TPX2 (amino acids 1–43) known to be sufficient for binding to and activating AURKA (Bayliss et al., 2003) and that is not degradable at mitotic exit. We overexpressed TPX2(1–43)–CFP in U2OS and FZR1^KO^ cells and monitored AURKA and pT288-AURKA levels during mitotic exit (Fig. 4). We found that persistence of TPX2(1–43) through mitotic exit stabilized AURKA protein in U2OS cells (Fig. 4A,B) as previously described (Giubettini et al., 2011). As expected, TPX2(1–43) expression showed no effect on AURKA levels in FZR1^KO^ cells (Fig. 4C,D). Importantly, we found a marked effect of TPX2(1–43) expression in stabilizing the pT288-AURKA signal during mitotic exit in both parental U2OS and FZR1^KO^ cells (Fig. 4A–D). We concluded that loss of interaction with TPX2 is the rate-limiting step in inactivation of AURKA at mitotic exit. We also found that inhibition of the candidate phosphatase PP1 after mitotic exit stabilized pT288 signal (Fig. 4E), consistent with the conclusion that deactivation rather than destruction controls AURKA activity during mitotic exit.

**Fig. 5.**
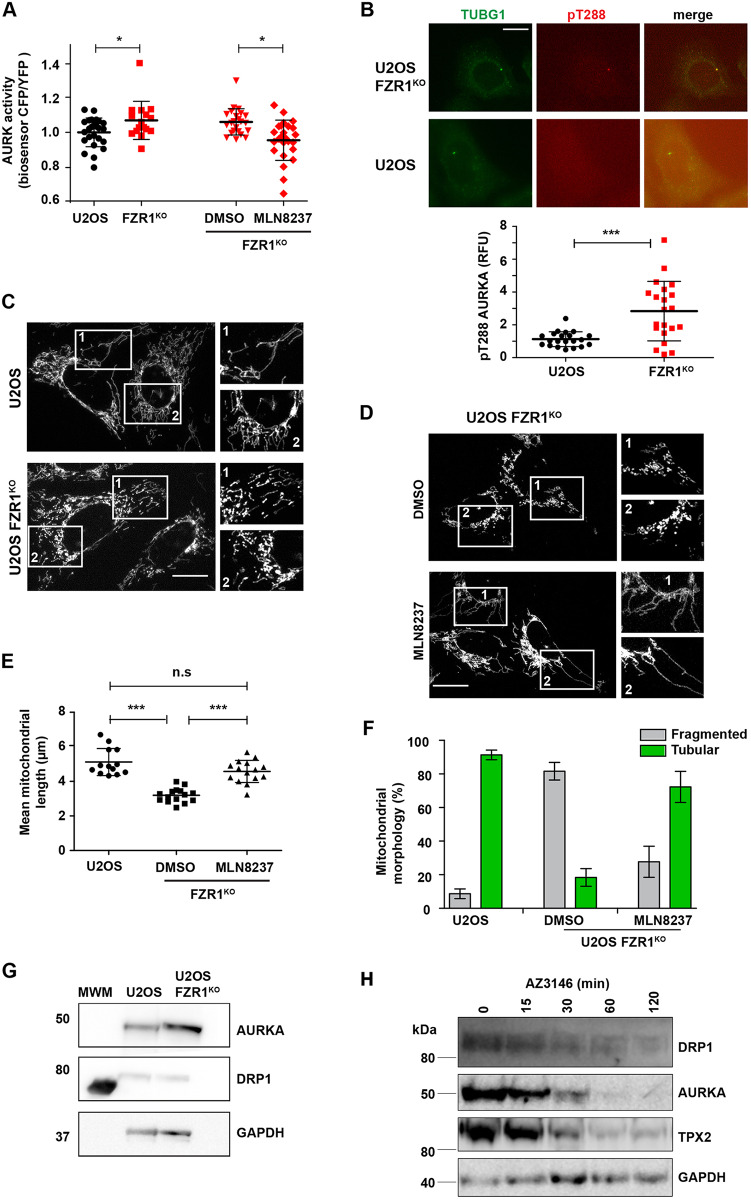
**Destruction of AURKA by APC/C–FZR1 is required to control interphase activity of AURKA.** (A) The FRET biosensor reveals raised Aurora kinase activity in interphase FZR1^KO^ cells. Cells were synchronized in G1/S using double thymidine block and treated (or not) for 3 h with 100 nM AURKA inhibitor MLN8237. Scatter plots show CFP/YFP emission ratios with mean±s.d. from individual cells in U2OS and FZR1^KO^ populations (*n*=20; *P*<0.05, Student's *t*-test) and are representative of two independent experiments. (B) pT288 staining in fixed cells synchronized at G1/S, and scatter plots of centrosomal pT288 signal quantified as in Fig. 1, show that pT288 can be detected at centrosomes of G1/S FZR1^KO^ cells, but not at centrosomes of parental U2OS cells. *n*≥21, ***P≤0.0001 (Student's *t*-test). RFU, relative fluorescence units. Scale bar: 10 μm. (C–F) Mitochondria are over-fragmented in interphase FZR1^KO^ cells in an AURKA-sensitive manner. U2OS and FZR1^KO^ cells were synchronized in G1/S then stained with MitoTracker™ and imaged live (C). FZR1^KO^ cells were treated with DMSO or 100 nM MLN8237 for 3 h (D). Numbered boxes indicate the regions shown in magnified images. Scale bars: 10 μm. (E) Quantitative analyses of fragmented mitochondria length was carried out as described in the Materials and Methods and are presented as scatter plots with mean±s.d. indicated (*n*≥15 cells; ***P<0.001 by two-tailed Mann–Whitney *U*-test), whilst (F) percentages of tubular versus fragmented morphologies were calculated using MicroP software and are presented as mean±s.d. (*n*≥15). (G,H) Immunoblotting U2OS and FZR1^KO^ cells shows that DRP1 levels, unlike those of AURKA, are not altered in G1/S FZR1^KO^ cells (G), even though DRP1 undergoes modest degradation during mitotic exit (H). GAPDH is shown as a loading control. MWM indicates molecular weight marker lane. Blots shown in G,H are representative of two independent experiments.

If FZR1-mediated destruction of AURKA is not required for its inactivation at mitotic exit, then what is it for? Our FRET recordings revealed a small but significant increase in Aurora kinase activity in FZR1^KO^ cells over the activity in parental U2OS cells, following mitotic exit (Fig. 3D). Using both the AURKA biosensor and a previously developed AURKB biosensor (Fuller et al., 2008), along with pT288 staining, we examined Aurora kinase activity in cells arrested at the G1/S boundary by double thymidine block. The AURKA biosensor revealed increased Aurora kinase activity in FZR1^KO^ cells, which was abolished by treatment with MLN8237 (Fig. 5A) but insensitive to low doses of AZD1152 inhibitor (20–30 nM) sufficient to abolish AURKB-specific activity (Fig. S4). Because the AURKB sensor is insensitive to MLN8237 at the dose used (Fig. S5), we concluded that there is increased AURKA activity in interphase in FZR1^KO^ cells. Consistent with this conclusion, we observed pT288 signal at centrosomes in interphase FZR1^KO^ but not parental U2OS cells (Fig. 5B).

Next, we explored how FZR1 affects known AURKA functions in interphase. We, and others, have previously described a role for AURKA, at physiologically relevant levels of expression, in promoting mitochondrial fission during interphase (Bertolin et al., 2018; Grant et al., 2018), an observation thought to be significant to cellular metabolism given the intimate link between mitochondrial morphology and function (Scott and Youle, 2010). We hypothesized therefore that one consequence of increased interphase AURKA activity in FZR1^KO^ cells would be increased mitochondrial fragmentation, and indeed mitochondrial fragmentation following FZR1 siRNA treatment of HeLa cells has previously been described (Horn et al., 2011). We compared the mitochondrial networks in parental U2OS and FZR1^KO^ cells arrested at the G1/S boundary, when the mitochondria tend to form large interconnected mitochondrial networks (Mitra et al., 2009; Salazar-Roa and Malumbres, 2017). We found that the mitochondrial network is highly fragmented in FZR1^KO^ cells derived from two independent CRISPR/Cas9 clones, and in a manner dependent on AURKA activity (Fig. 5C–F; Fig. S5). We measured reduced mean mitochondrial lengths in FZR1^KO^ cells (Fig. 5E), and classified mitochondria according to morphological subtypes, finding a significant increase in the percentage of fragmented globules and significantly reduced tubular morphology in FZR1^KO^ cells (Fig. 5F). Horn et al. (2011) identified the fission factor dynamin-related protein-1 (DRP1, also known as DNM1L) as the target of FZR1 required for its effect on mitochondrial morphology. Here we found that inhibition of AURKA activity completely rescued mitochondrial length and morphology in FZR1^KO^ cells (Fig. 5E,F; Fig. S5), consistent with data showing AURKA to be an upstream regulator of DRP1 (Bertolin et al., 2018; Kashatus et al., 2011). By contrast, treatment of FZR1^KO^ cells with an AURKB-specific inhibitor had no effect on mitochondrial length (Fig. S5). We found no alteration in DRP1 levels in FZR1^KO^ cells (Fig. 5G) although, in agreement with Horn et al. (2011), we observed loss of DRP1 during mitotic exit in parental cells (Fig. 5H) and suggest that DRP1 could be a substrate for APC/C–CDC20 as well as APC/C–FZR1, or another ubiquitin ligase. We concluded that APC/C–FZR1 regulates mitochondrial dynamics by preventing AURKA reactivation in interphase.

**Fig. 6.**
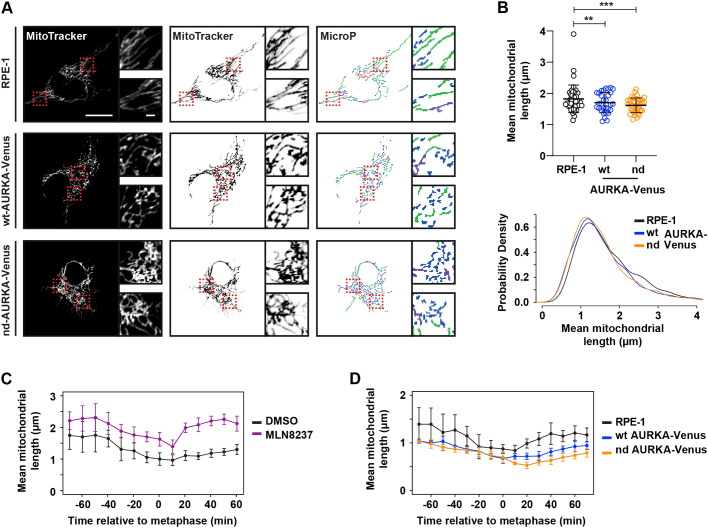
**Undegraded AURKA at mitotic exit inhibits reassembly of the interphase mitochondrial network.** (A,B) RPE-1, RPE-1 WT-AURKA–Venus and non-degradable version (nd, Δ32–66) cell lines were stained with MitoTracker™ and imaged 24 h after induction of AURKA transgene expression. Acquired images were analysed with MicroP (A) and subjected to mitochondria length quantifications (B). Dashed boxes indicate regions shown in magnified images. Inverted images (A, middle) and output of MicroP length analysis (A, right) are shown. Scatter plots show mean±s.d. for mean mitochondrial lengths from 30 mitochondria per cell in 18–24 cells per condition from two experimental replicates (upper panel). *P*-values are calculated from raw measurements using a Mann–Whitney test: **P<0.001; ***P<0.0001; *n*≥600. Lower panel shows kernel density plots of raw data. Scale bars: 10 μm in large panels, 1μm in inset panels. (C) RPE1 cells treated with either DMSO or 100 nM MLN8237 were stained with MitoTracker™ and filmed live as they progressed through mitosis. Mean±s.d. mitochondrial length over time is plotted for *n*≥6 cells per condition in two experimental replicates. (D) RPE-1 WT-AURKA–Venus and nd-AURKA–Venus cells were stained with MitoTracker™, then filmed and analysed as in C. Data are mean±s.d. of *n*≥5 cells.

To further test our hypothesis that mitochondrial fragmentation is a direct response to undegraded AURKA, we tested whether the introduction of nondegradable (nd, Δ32–66) or wild-type (WT) versions of AURKA would show differential effects on mitochondrial dynamics (Fig. 6). We used stable RPE1-FRT/TO cell lines expressing either WT-AURKA–Venus or nd-AURKA–Venus under tetracycline control. Induction of WT-AURKA–Venus caused increased mitochondrial fragmentation in unsynchronized cells, as previously described (Grant et al., 2018), but nd-AURKA–Venus had a greater effect (Fig. 6A,B). Importantly, this mitochondrial phenotype was independent of any effect of AURKA on microtubule dynamics that might contribute to subcellular distribution of mitochondria (Fig. S6). We then tracked individual cells through mitotic exit in the presence of MitoTracker™ stain to label mitochondria. Treatment with MLN8237 interfered with mitochondrial fragmentation at mitosis, as expected from previously published findings (Kashatus et al., 2011) (Fig. 6C). In the presence of overexpressed AURKA–Venus we found that mitochondria were excessively fragmented as cells progressed out of mitosis into G1 phase, and that this effect was exacerbated in cells expressing nd-AURKA–Venus compared to cells expressing WT-AURKA–Venus (Fig. 6D). We concluded that undegraded AURKA is able to inhibit reassembly of the interphase mitochondrial network, and that AURKA is a direct target of FZR1 in regulating mitochondrial dynamics.

## DISCUSSION

AURKA activity increases in preparation for mitosis in parallel with increases in the protein level, and both progressively drop from anaphase onwards. This has led to the expectation that destruction contributes to regulation of AURKA activity at mitotic exit (Afonso et al., 2017; Floyd et al., 2008). In this study, however, we found that loss of AURKA activity and destruction of the protein are uncoupled. Lack of the APC/C co-activator FZR1 completely stabilized AURKA levels but did not affect the timing of AURKA inactivation during mitotic exit and into the subsequent G1 phase, as measured using pT288 reactivity or a novel FRET biosensor for AURKA activity that we characterize in this study. Therefore, AURKA destruction is not required to regulate the timing of AURKA inactivation after mitosis.

Instead, we found that FZR1 suppresses AURKA-dependent activity of the FRET biosensor in interphase. In the absence of FZR1 we speculate that the timing of reactivation of AURKA depends on the changing balance of AURKA activators and phosphatases during G1 phase. Although a low level of pT288 was detectable at centrosomes by IF in G1-phase FZR1^KO^ cells, since there are routes to generating active AURKA that do not depend on autophosphorylation – for example, through interaction with Nucleophosmin/B23 or TACC3 (Burgess et al., 2015; Reboutier et al., 2012) – it is likely that basal AURKA activity in interphase is dependent on AURKA levels in the cell, and is calibrated in each cell cycle by mitotic destruction of the AURKA pool. Such multiple ‘opportunities’ for AURKA activation could underlie the strong association of AURKA overexpression with cancer, particularly where co-overexpressed with an activating partner such as TPX2 (Asteriti et al., 2010), and underscore the importance of ubiquitin-mediated destruction of AURKA in suppressing excess AURKA activity in interphase.

We investigated further the route to inactivation of the mitotic pool of AURKA. We found, as expected, that pT288 dephosphorylation during mitotic exit could be triggered through loss of interaction with TPX2. TPX2 has been shown to protect AURKA from negative regulation by PP1 (Bayliss et al., 2003; Eyers et al., 2003; Katayama et al., 2001; Tsai et al., 2003). Indeed, whereas PP6 has been shown to act on the AURKA–TPX2 complex to limit AURKA activity in spindle assembly, PP1 can act only on ‘free’ AURKA (Zeng et al., 2010). We found that overexpression of the TPX2 (1–43) peptide that is sufficient to activate AURKA, and which is not degraded at mitotic exit, delayed the timing of kinase inactivation.

Our data indicate that the ‘release’ of AURKA to PP1-mediated inactivation could depend on APC/C–CDC20-mediated destruction of TPX2. We cannot exclude that ubiquitination of TPX2 would be enough to abrogate interaction, or that loss of interaction would be an indirect consequence of APC/C–CDC20 activity. However, our idea is consistent with the early degradation of TPX2 that we observe, and is in agreement with studies indicating TPX2 as a major substrate for APC/C at mitotic exit (Min et al., 2014; Singh et al., 2014) and numerous observations that amplification of TPX2 is the rate-limiting event for increased AURKA activity associated with tumorigenesis (Asteriti et al., 2010; Shah et al., 2019). One interesting conclusion to be drawn from our model is that, although AURKA destruction during mitotic exit is a FZR1-dependent event, AURKA inactivation during mitotic exit is dependent on CDC20, along with all of the other known critical events of mitotic exit. This helps to explain why mitotic exit occurs essentially normally in the absence of FZR1. Indeed, FZR1 is not expressed during early embryonic cell cycles, and AURKA activity is regulated by activation and inactivation, instead of periodic proteolysis (Littlepage and Ruderman, 2002). Throughout development, AURKA destruction therefore remains ‘decoupled’ from mitosis in a manner that contrasts with most other substrates of APC/C–FZR1 that can also be targeted by APC/C–CDC20 (Lindon et al., 2015). We infer that the unusual relationship between APC/C and Aurora kinases preserves a pool of potential Aurora kinase activity with critical interphase functions, regulatable through proteostasis.

So, what are the FZR1-sensitive AURKA-dependent events of interphase? Previous studies have shown that AURKA activity acts as an upstream regulator of mitochondrial morphology through RALA-dependent and -independent pathways (Bertolin et al., 2018; Kashatus et al., 2011). Mitochondria are highly dynamic organelles that undergo constant fission and fusion to modulate connectivity of the mitochondrial network, allowing the cell to respond to metabolic demands and maintain a healthy mitochondrial network. Increased rates of fission occur in preparation for mitosis so that mitochondrial fragments can be equally distributed between daughter cells, and reassembly of the mitochondrial network is a critical step in the post-mitotic reconstitution of the interphase state. Here we show that the presence of non-degraded AURKA delays reassembly of the mitochondrial network after cell division. Whilst there is increasing evidence that the dynamic state of mitochondria contributes to the overall metabolic state of the cell, our understanding of how this impacts progression in the cell cycle remains limited (Mitra et al., 2009; Salazar-Roa and Malumbres, 2017). Our study provides new evidence that control of AURKA activity in interphase by APC/C–FZR1 is a critical parameter in the regulation of mitochondrial dynamics.

## MATERIALS AND METHODS

### Generation of U2OS FZR1^−/−^ knockout cell line (FZR1^KO^)

Two guide RNAs (5′-GACGTCCGATTGGAACGGCG-3′ and 5′-GCCCTGCCTCGCCATGGACC-3′) targeting the first exon of FZR1 were cloned into the AIO-GFP nickase vector, a gift from Steve Jackson (Wellcome Trust/Cancer Research UK Gurdon Institute, Cambridge, UK; Addgene plasmid # 74119) (Chiang et al., 2016).

U2OS cells were transfected with 2 µg of plasmid by electroporation using the Neon Transfection System according to the manufacturer's instructions (Thermo Fisher Scientific). 48 h after transfection, cells were sorted based on GFP fluorescence into 96-well plates at a single-cell-per-well density for clonal expansion. Single-cell clones were validated for loss of FZR1 by sequencing the genomic locus, immunoblotting and live-cell imaging of APC/C substrates. We tested that U2OS parental and FZR1^KO^ cells have identical DNA content and are mycoplasma-free.

### Cell culture, synchronization and drug treatments

U2OS and FZR1^KO^ cells were cultured in DMEM (Thermo Fisher Scientific) supplemented with 10% FBS, 200 µM GlutaMAX-1 (Thermo Fisher Scientific), 100 U/ml penicillin, 100 µg/ml streptomycin, and 250 ng/ml fungizone at 37°C with 5% CO2. For mitotic exit synchronizations, cells were collected in mitosis by treatment for 12 h with 10 μM STLC (S-Trityl-L-cysteine; Tocris Bioscience) to trigger the spindle assembly checkpoint (SAC) and then released by treatment with 10 μM AZ3146 (Generon, Slough, UK), an inhibitor of the SAC kinase Mps1. Cells were synchronized at other cell cycle stages as follows. For G0, cells were starved for 48 h in DMEM without serum. For G1, G0-arrested cells were released into serum-containing medium for 2 h. For G1/S, cells were incubated with medium containing 2 mM thymidine for 16 h, washed with PBS, released into regular medium for 12 h, and then incubated in medium containing 2 mM thymidine for 15 h. S-phase cells were prepared by releasing G1/S phase cells into regular medium minus thymidine for 5 h. To prepare an M-phase cell population, cells were incubated with 10 µM STLC for 12 h. Mitotic cells were then collected by shake-off.

Aurora kinase inhibitors MLN8237 (Stratech, Ely, UK), MK5108 (Axon Medchem, Groningen, Netherlands), ZM447439 (Generon) and AZD1152-HPQA (Sigma-Aldrich UK) were used at the doses indicated.

RPE-1 cells, and RPE-1 FRT/TO cell lines expressing WT-AURKA–Venus and nd-AURKA–Venus were cultured as previously described (Grant et al., 2018).

### Plasmids and transient transfection

pVenus-N1-AURKA, AURKA-Δ32–66 (Floyd et al., 2008; Littlepage and Ruderman, 2002) and TPX2(1–43) (Giubettini et al., 2011) have been described previously in the publications cited.

The non-targeted AURKB FRET biosensor was a kind gift from Michael Lampson (University of Pennsylvania, PA, USA; Fuller et al., 2008).

The AURKA-directed FRET biosensor was created as follows. We modified a FRET construct backbone containing mTurquoise and mVenus sequences in the pECFP-C1 Clontech vector [construct ‘F36’ (Piljić et al., 2011), a kind gift from Carsten Schultz, Oregon Health and Science University, Portland, OR, USA]. First, mVenus was replaced by YPet sequence using KpnI and BamHI restriction sites. The sequence of the FHA2 domain from ScRad53 was amplified by PCR and inserted between AgeI and MluI sites, introducing HindIII and EcoRI sites upstream of MluI. Finally, complementary oligonucleotides encoding the phosphorylation site Thr210 of PLK1 were annealed and inserted between EcoRI and MluI sites. The amino acid sequence used was *GGSGG*KVYDGERKKK**T**LCI. Note that an isoleucine was introduced at the +3 position for FHA2 binding. The final plasmid construct contains the following elements: NcoI-mTurquoise-BglII-AgeI-FHA2-HindIII-EcoRI-PLK1 T210 phosphorylation sequence-MluI-YPet-BamHI. Complete sequence is available upon request.

Cells were transfected using electroporation with Neon Transfection System (Invitrogen) using the following parameters: pulse voltage 1500 V, pulse width 10 ms, and 2 pulses total on the transfection device, according to the manufacturer's protocol.

### Immunoblotting

Cells were lysed in 1% Triton X-100, 150 mM NaCl, 10 mM Tris-HCl at pH 7.5, EDTA-free protease inhibitor cocktail (Roche) and PhosSTOP™ inhibitor for phosphatases (Sigma-Aldrich). After 30 min on ice, the lysate was centrifuged at 16,000 ***g*** for 10 min at 4°C. For immunoblotting, an equal amount of protein (20 μg) was loaded into SDS–PAGE 4–12% pre-cast gradient gels. Proteins were transferred to Immobilon-P or Immobilon-FL membranes (Merck Millipore, Darmstadt, Germany) using an XCell II Blot Module (Thermo Fisher Scientific) according to the manufacturer's instructions. Membranes were blocked in PBS containing 0.1% Tween-20 and 5% bovine serum albumin (BSA) then processed for immunoblotting. Primary antibodies for immunoblot were as follows: anti-AURKA mouse mAb (1:1000; Clone 4/IAK1, BD Transduction Laboratories), anti-phospho-Aurora A (Thr288)/Aurora B (Thr232)/Aurora C (1:1000; clone D13A11 XP rabbit mAb, Cell Signaling Technology), rabbit polyclonal anti-TPX2 antibody (1:1000; Novus Biological), anti-Cdh1 mouse mAb (1:50; gift from Tim Hunt and Julian Gannon, Cancer Research UK London Research Institute, London, UK), anti-CDC20 mouse mAb (1:1000; Santa Cruz sc13162), anti-AURKB rabbit polyclonal antibody (1:1000; Abcam ab2254), mouse monoclonal anti-cyclin B1 (1:1000; BD 554177), anti-DRP1 rabbit polyclonal (1:500; Bethyl Laboratories), rabbit polyclonal anti-tubulin (1:2000; Abcam ab6046), mouse mAb anti-vinculin (1:1000; clone hVIN-1, Sigma-Aldrich), rabbit anti-GFP (1:1000; 11814460001, Roche). Secondary antibodies used were HRP-conjugated, or IRDye 680RD- or 800CW-conjugated at 1:10,000 dilution for quantitative fluorescence measurements on an Odyssey Fc Dual-Mode Imaging System (LICOR Biosciences). Quantitative immunoblotting was carried out using IRDye 680RD and 800CW fluorescent secondary antibodies, scanned on an Odyssey Imaging System (LI-COR Biosciences).

### Immunofluorescence analysis

Cells were seeded at 2×10^4^ onto glass coverslips and then fixed with cold 100% methanol (−20°C), permeabilized with 0.5% Triton X-100 in PBS and incubated in 2% BSA, 0.2% Triton X-100 in PBS (blocking buffer) for 1 h at room temperature. Cells were incubated overnight with primary antibodies (rabbit anti-phospho-AURKA Thr288, Cell Signaling Technology, 1:50; mouse anti-γ-tubulin GTU-88, Sigma-Aldrich, 1:1000) diluted in blocking buffer at 4°C, then washed three times with blocking buffer and incubated with secondary antibodies at 1:1000 dilution. Alexa Fluor 488 anti-mouse and Alexa Fluor 568 anti-rabbit (Thermo Fisher Scientific) were used as the secondary antibodies. DNA was stained with DAPI. Coverslips were mounted with Prolong Gold antifade reagent. Epifluorescence stacks were acquired using 500 nm *z* step with 2×2 bin using appropriate filter sets and a 40× NA 1.3 oil objective. The best in-focus images were selected and integrated intensities were measured using ImageJ (http://rsb.info.nih.gov/ij/; National Institutes of Health, Bethesda, MD).

### Mitochondrial imaging and analysis

Cells were seeded at 2×10^4^ onto eight-well plastic-bottom slides (Ibidi GmbH, Martinsried, Germany) for live-cell imaging. To stain the mitochondria, the cells were incubated with 100 nM MitoTracker™ Red CMXRos (M7512, Thermo Fisher Scientific) for 15 min, which was then replaced with L-15 medium (Thermo Fisher Scientific) supplemented with FBS. Epifluorescence images were acquired with 40× NA 1.3 oil objective on an Olympus IX81 motorized inverted microscope (Olympus Life Science, Southend-on-Sea, UK). The automated imaging platform included PE4000 LED illumination source (CoolLED, Andover, UK), Retiga R6 CCD camera (QImaging, Birmingham, UK), motorized stage (Prior Scientific, Cambridge, UK) and 37°C incubation chamber (Solent Scientific, Segensworth, UK), all controlled by Micro-Manager (Edelstein, 2014). Images were collected with 2×2 bin applied, exported as tiff files and analysed using MicroP (Grant et al., 2018; Peng et al., 2011). For mitochondrial length analyses, each datapoint represents the mean value of 30 mitochondrial fragments per cell.

### Timelapse imaging and FRET quantification

Cells were imaged in L-15 medium with 10% FBS at 37°C using an automated epifluorescence imaging platform composed of Olympus IX83 motorized inverted microscope, Spectra-X multi-channel LED widefield illuminator (Lumencor, Beaverton, OR, USA), Optospin filter wheel (Cairn Research, Faversham, UK), CoolSnap MYO CCD camera (Photometrics, Tucson, AZ, USA), automated XY stage (ASI, Eugene, OR, USA) and climate chamber (Digital Pixel, Brighton, UK) and controlled using Micro-Manager. FRET imaging was performed using a 40× NA 0.95 objective and ECFP/EYFP/mCherry beamsplitter (Chroma, Bellows Falls, VT, USA) for ratiometric comparison of CFP and YFP emission upon excitation of CFP. ImageJ software (National Institutes of Health) was used to quantify CFP and YFP signal across the whole cell, and AURKA activity was expressed as CFP/YFP ratio (1/FRET).

### Statistical analysis

Data analyses were performed using GraphPad 6.01 (San Diego, CA, USA). Results were analysed with a Student's *t*-test or Mann–Whitney *U*-test (non-parametric) as indicated in figure legends. Significant results are indicated as *P*<0.05 (*), *P*≤0.01 (**) or *P*≤0.001 (***). Values are stated as the mean±s.d.

## Supplementary Material

Supplementary information

Reviewer comments
