# Supplementary material for: AURKA destruction is decoupled from its activity at mitotic exit but is essential to suppress interphase activity
Source: J Cell Sci. 2020 Jun 16;133(12):jcs243071. doi: 10.1242/jcs.243071 (PMC7328152; doi:10.1242/jcs.243071)
Supplement: Supplementary information [file joces-133-243071-s1.pdf]

SUPPLEMENTARY FIGURES

AURKA destruction is decoupled from its activity at mitotic exit but essential to suppress interphase activity.
Abdelbaki et al.

Figure S1

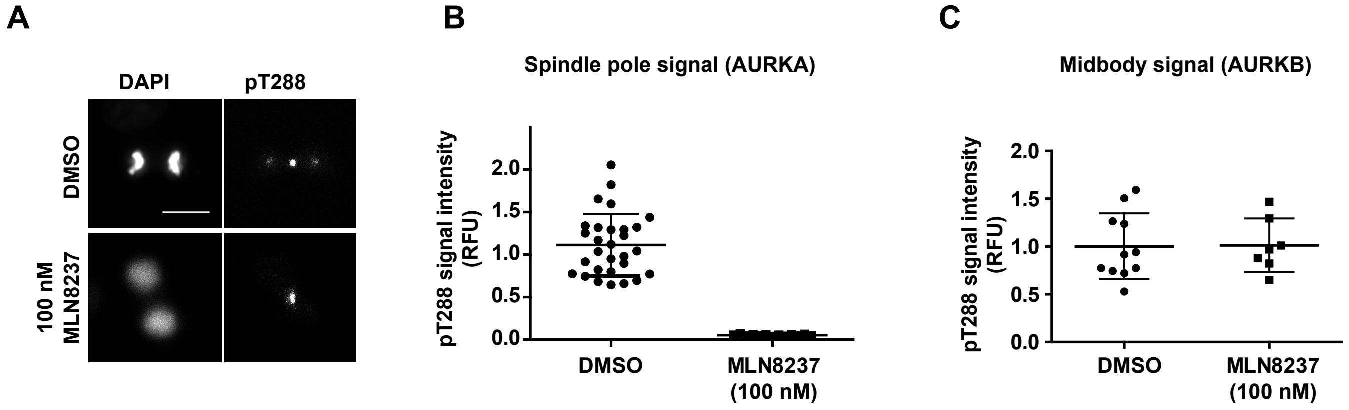

Figure S1: Specificity of pT288-AURKA signal in mitotic cells. Immunofluorescence analysis of mitotic U2OS cells fixed and stained with antibody against pT288-AURKA shows signal at both spindle poles and midbody (**A**). pT288 signal at spindle poles and midbody was measured in cells treated for 3 hours prior to fixation with 100 nM AURKA inhibitor MLN8237 or DMSO control (**B**, **C**). Spindle pole signal was ablated following MLN8237 treatment and is therefore specific for AURKA activity (**B**) whereas midbody signal is refractory to MLN8237 inhibition and attributed to recognition of pT232-AURKB epitope (**C**). Scatter plots show distributions with mean ± S.D. of pT288 signal intensity measurements expressed as background-corrected mean pixel values in a ROI at spindle pole or midbody, normalized to the mean value of the control (DMSO-treated) population. RFU, Relative Fluorescence Units. Size bar, 10 μM.

Journal of Cell Science • Supplementary information

Figure S2

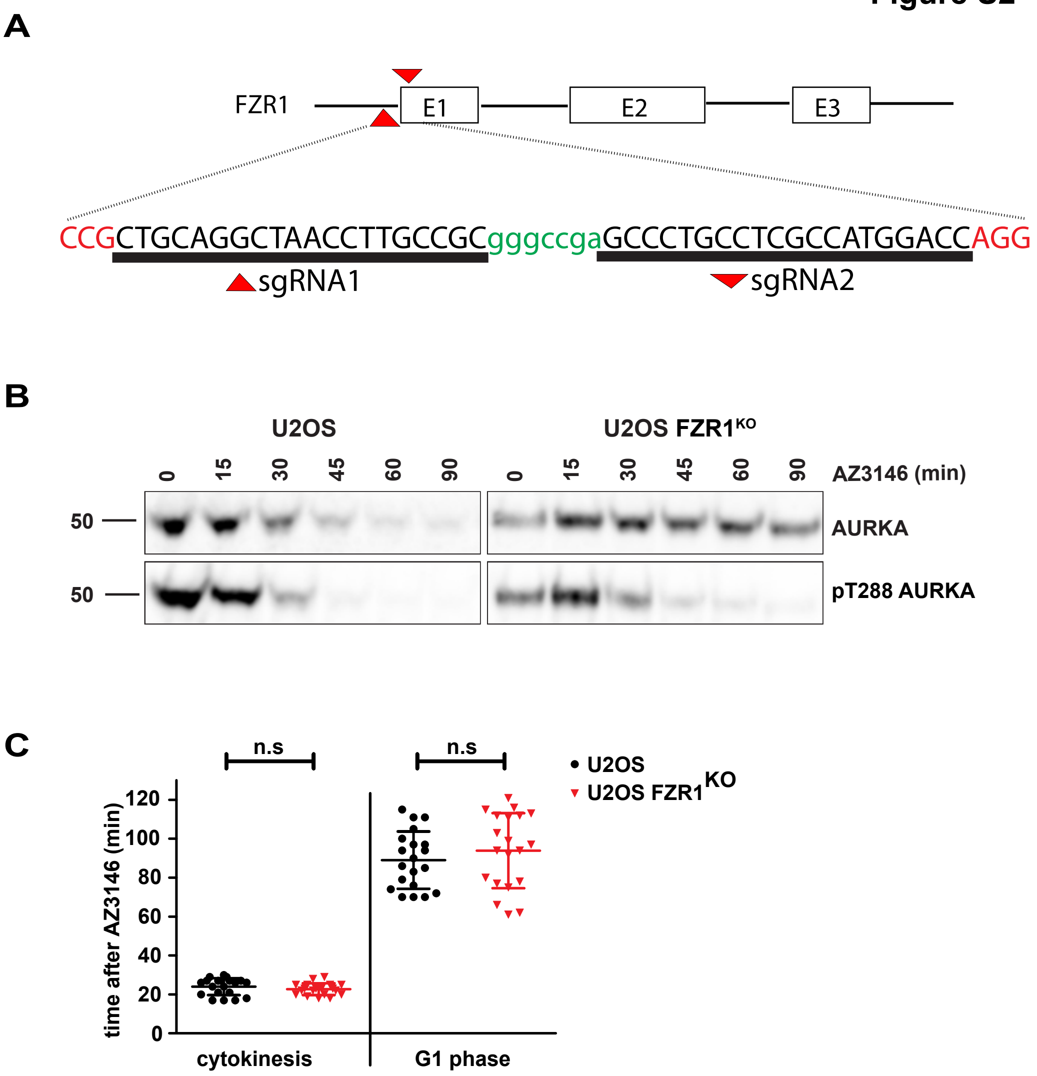

Figure S2: Regulation of AURKA during mitotic exit in FZR1^{KO} cells. A Schematic showing guide RNAs used to generate CRISPR/Cas9 knockout of FZR1. **B** Dynamics of AURKA degradation and inactivation in a second clone of FZR1^{KO} cells. Extracts were prepared and processed as in Figure 2C and blotted with the antibodies indicated. **C** Kinetics of forced mitotic exit in parental U2OS and FZR1^{KO} cells. Cells synchronized according to the protocol used for Figure 2C immunoblots were pre-incubated for one hour with 10uM sir-DNA, then filmed by timelapse microscopy to score cells for cytokinesis timing (onset of cortical contractility) and return to interphase (decondensed chromosomes, nuclear envelope reformed, cells adhered).

Journal of Cell Science • Supplementary information

Figure S3

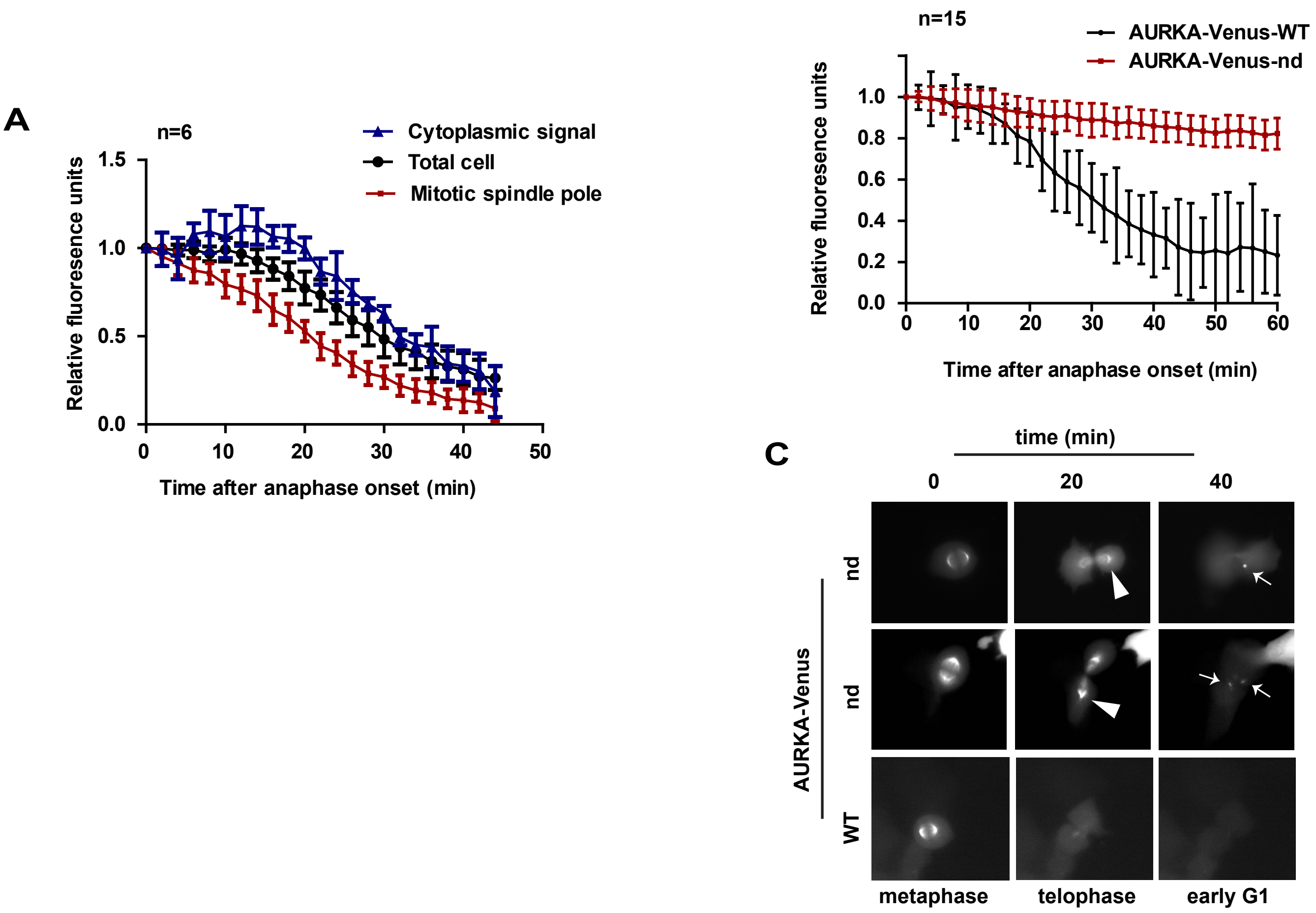

Figure S3: FZR1-dependent degradation of AURKA at spindle poles. A-C Undegraded AURKA persists at spindle poles: RPE-1 FRT/TO cells expressing tet-inducible wild-type (WT) or non-degradable (Δ33-64, nd) AURKA-Venus were subjected to timelapse fluorescence imaging during mitosis, and fluorescence levels quantified as readout for mitotic exit degradation of AURKA. **A** Separate quantification on spindle poles and in the cytoplasm shows robust loss of AURKA-Venus-WT from spindle poles correlates with degradation of the protein (loss of total cell signal), n ≥ 6. **B** AURKA-Venus-nd is stable during the mitotic exit window when WT-AURKA-Venus is degraded (n ≥ 15) and (**C**) remains strongly associated with spindle poles in telophase (arrowheads). **C** AURKA-Venus-nd can still be detected at centrosomes in early G1 phase (arrows), at a time when AURKA-Venus-WT is no longer detectable.

Journal of Cell Science • Supplementary information

J. Cell Sci.: doi:10.1242/jcs.243071: Supplementary information

Figure S4

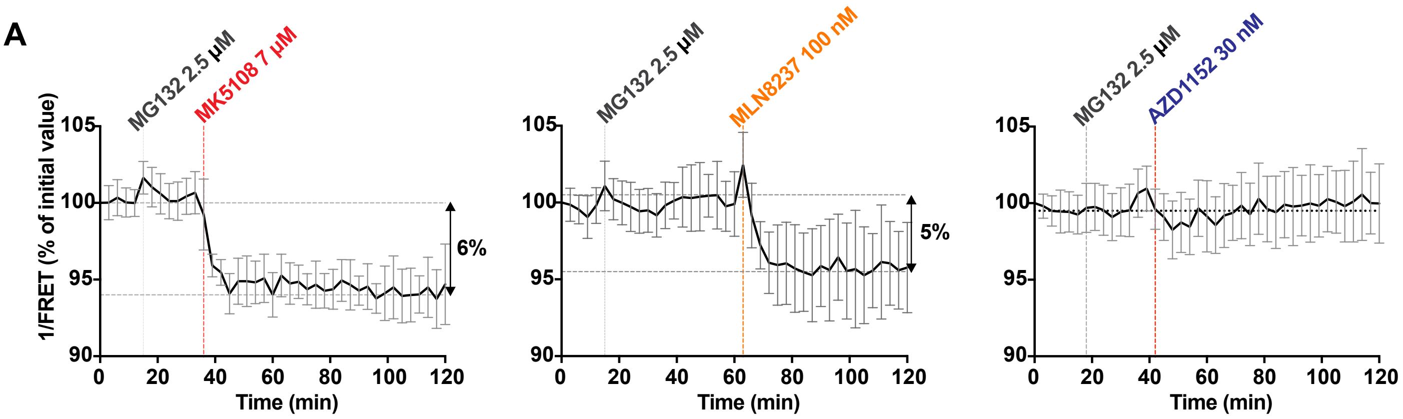

	[inhibitor]	mitotic activity inhibition	number of cells analysed
AURKA inhibitor	MK5108 7 µM	6%	13
	MLN8237 200 nM	6%	16
	MLN8237 100 nM	5%	13
AURKB inhibitor	ZM447439 2 µM	2%	12
	AZD1152 500 nM	2%	22
	AZD1152 30 nM	< 1%	18

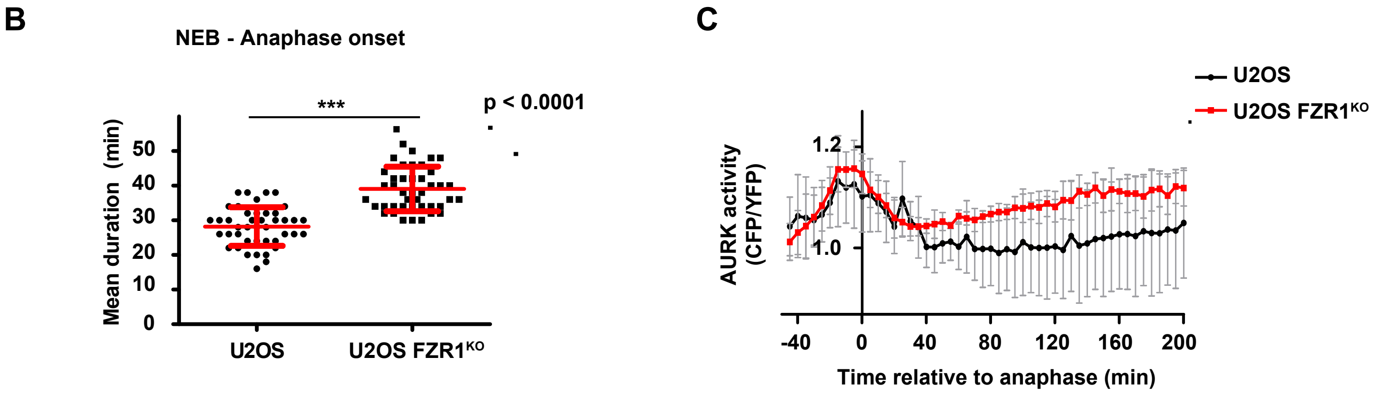

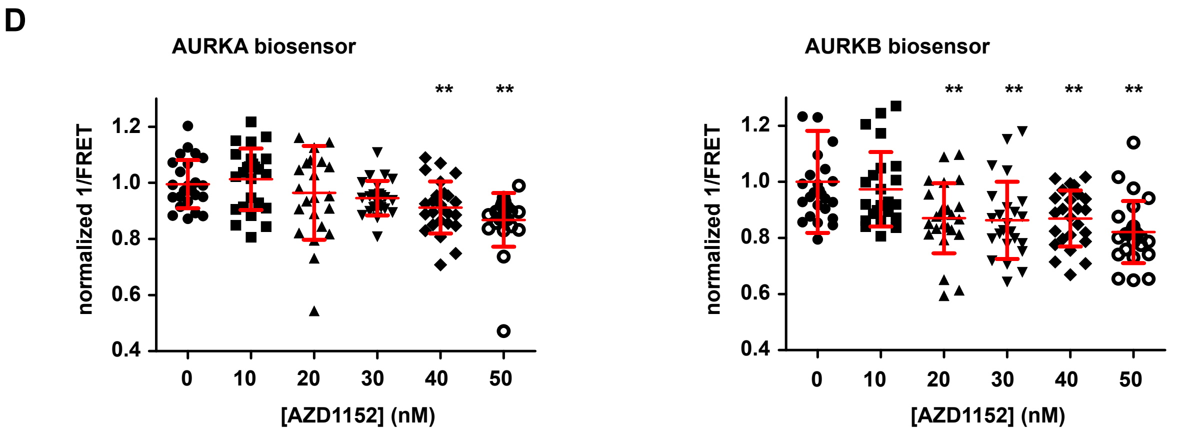

Figure S4: Measuring Aurora kinase activity with a FRET-based biosensor.

A U2OS cells transfected with the AURKA-directed biosensor from a population synchronized by release from double thymidine block were arrested in mitosis by treatment with MG132, then treated with AURKA- or AURKB-specific inhibitors. Mean activity values (1/FRET) for mitotic cells

Journal of Cell Science • Supplementary information

undergoing different drug treatments are shown in the traces (upper panels) and results from a number of different experiments summarized in the table below. Biosensor activity was strongly inhibited by treatment with MLN8237 or MK5108, specific inhibitors of AURKA, and partially inhibited by two inhibitors of AURKB, AZD1152 and ZM447439. **B** Mitotic exit is delayed by approximately 10 min in FZR1^{KO} cells. Times from NEB to anaphase in individual cells are shown as scatter plots with mean values ± S.D., n > 40, $p < 0.001$ by Students *t*-test. **C** AURKA-directed biosensor activity (data from Figure 3D) *in silico* synchronized to anaphase onset to take account of different timings shown in **B**. **D** Activity of AURKA-directed biosensor in interphase FZR1^{KO} cells is insensitive to 30 nM dose of AZD1152 that is sufficient to inhibit activity of an AURKB-directed sensor. FZR1KO cells were arrested at G1/S by application of double thymidine block and treated for 3 hours with AZD1152 at indicated doses. 1/FRET values calculated for individual cells and normalized against the population mean are shown as scatter plots with mean ± S.D. indicated foreach condition. **, $p < 0.001$, Mann-Whitney U-test.

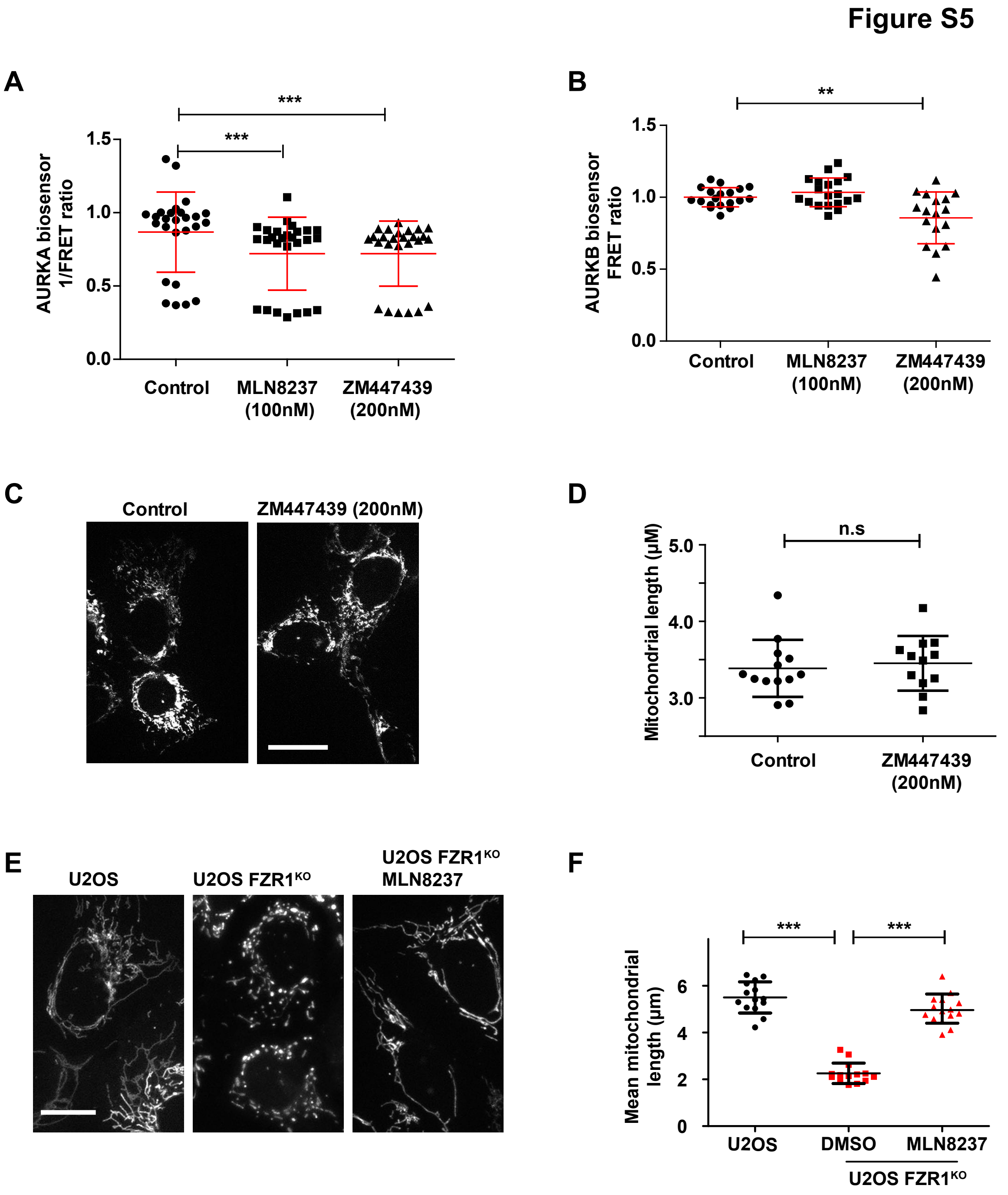

Figure S5: Regulation of mitochondrial fragmentation in FZR1^{KO} cells depends on AURKA. A-B FZR1^{KO} cells transfected with either AURKA- or AURKB-directed cytoplasmic biosensors were synchronized at the G1/S boundary as in Figure 4 and challenged for 3 hours with AURKA-specific inhibitor MLN8237 or AURKB-specific inhibitor ZM447439 at the indicated doses. FRET values are presented as in Figure S4D. **A** The AURKA biosensor signal was sensitive to either inhibitor (n ≥ 25). **B** the AURKB biosensor responded to ZM447439 but was insensitive to MLN8237 (n ≥ 19) and we concluded that MLN8237 does not inhibit AURKB activity in FZR1^{KO} cells. **C, D** FZR1^{KO} cells were treated with 200 nM ZM447439 for 3 hours then stained with MitoTracker® for measurement of mitochondrial fragment lengths. AURKB inhibition had no effect on mitochondrial fragmentation, as illustrated in representative panels shown in **C,** and in scatter plots (**D**) expressed as means of 30 mitochondrial fragment lengths per cell, n ≥ 13. **, $p < 0.01$; ***, $p < 0.001$; n.s. non-significant, by Mann-Whitney U-test. **E, F** AURKA activity-dependent fragmentation of mitochondria in a second FZR1^{KO} clone. Mitochondrial lengths were quantified in a replicate of the experiment shown in Figure 5E. ***, $p < 0.001$ by Mann-Whitney U-test.

Journal of Cell Science • Supplementary information

Figure S6

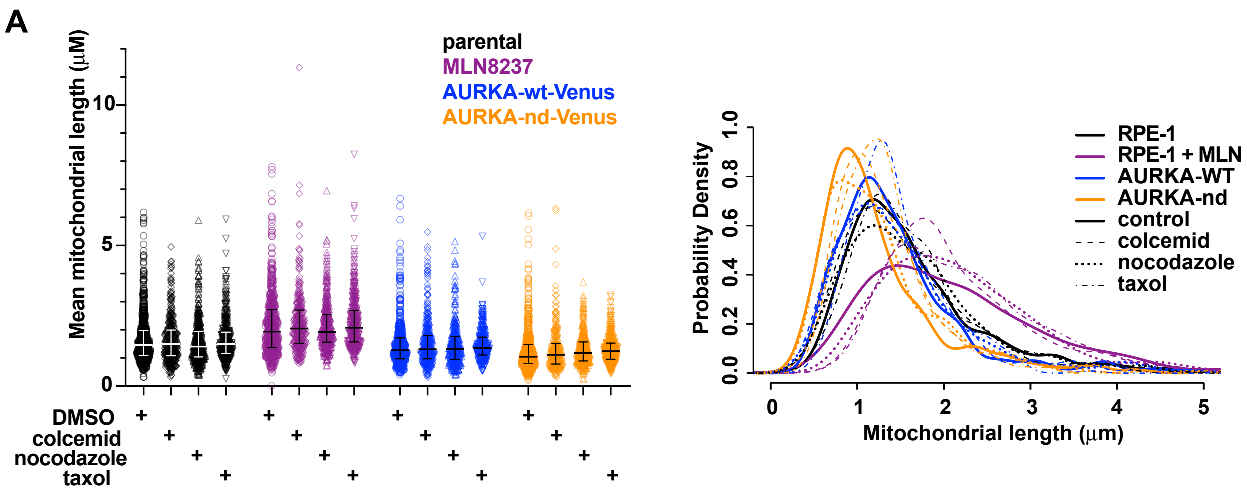

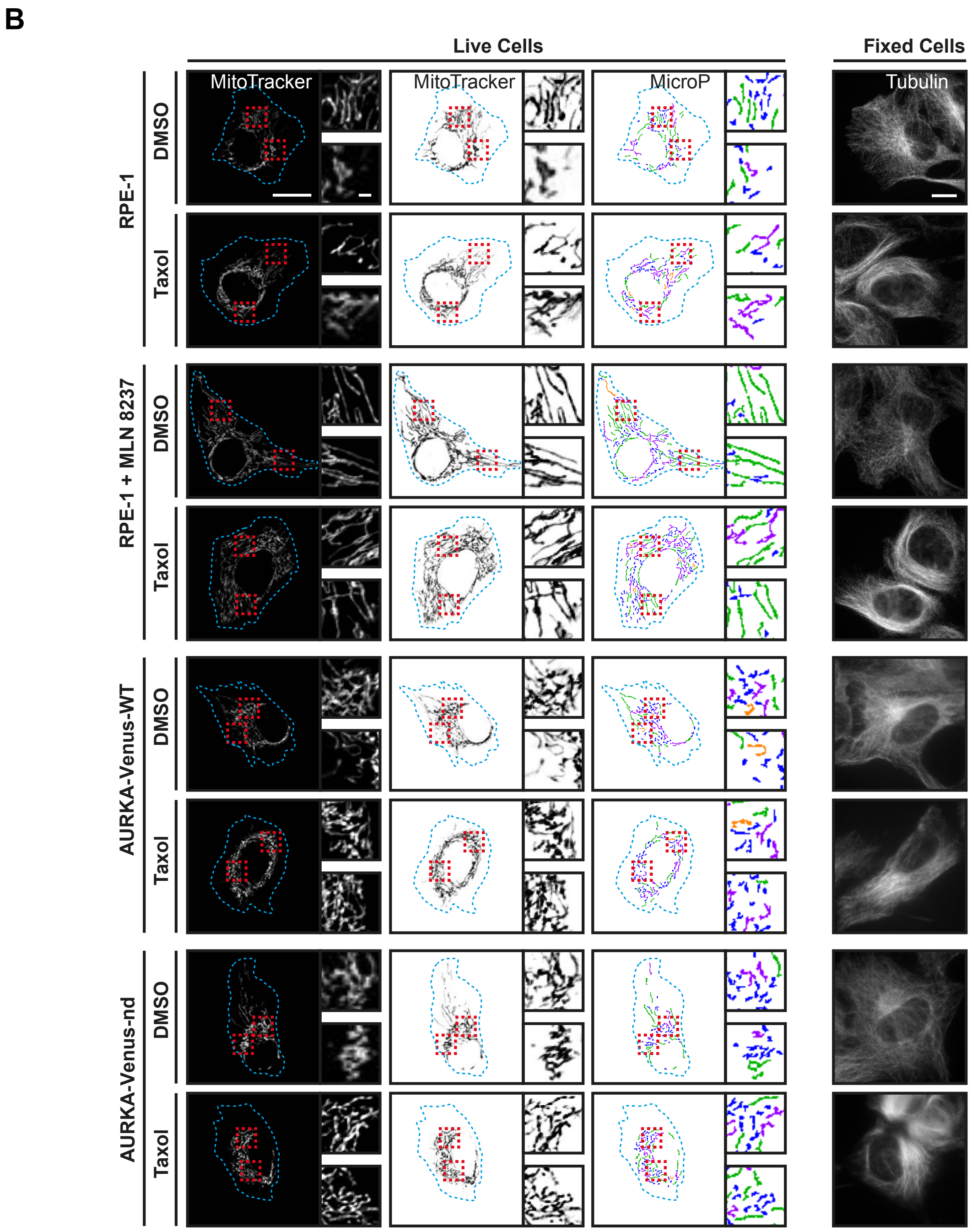

Journal of Cell Science • Supplementary information

C

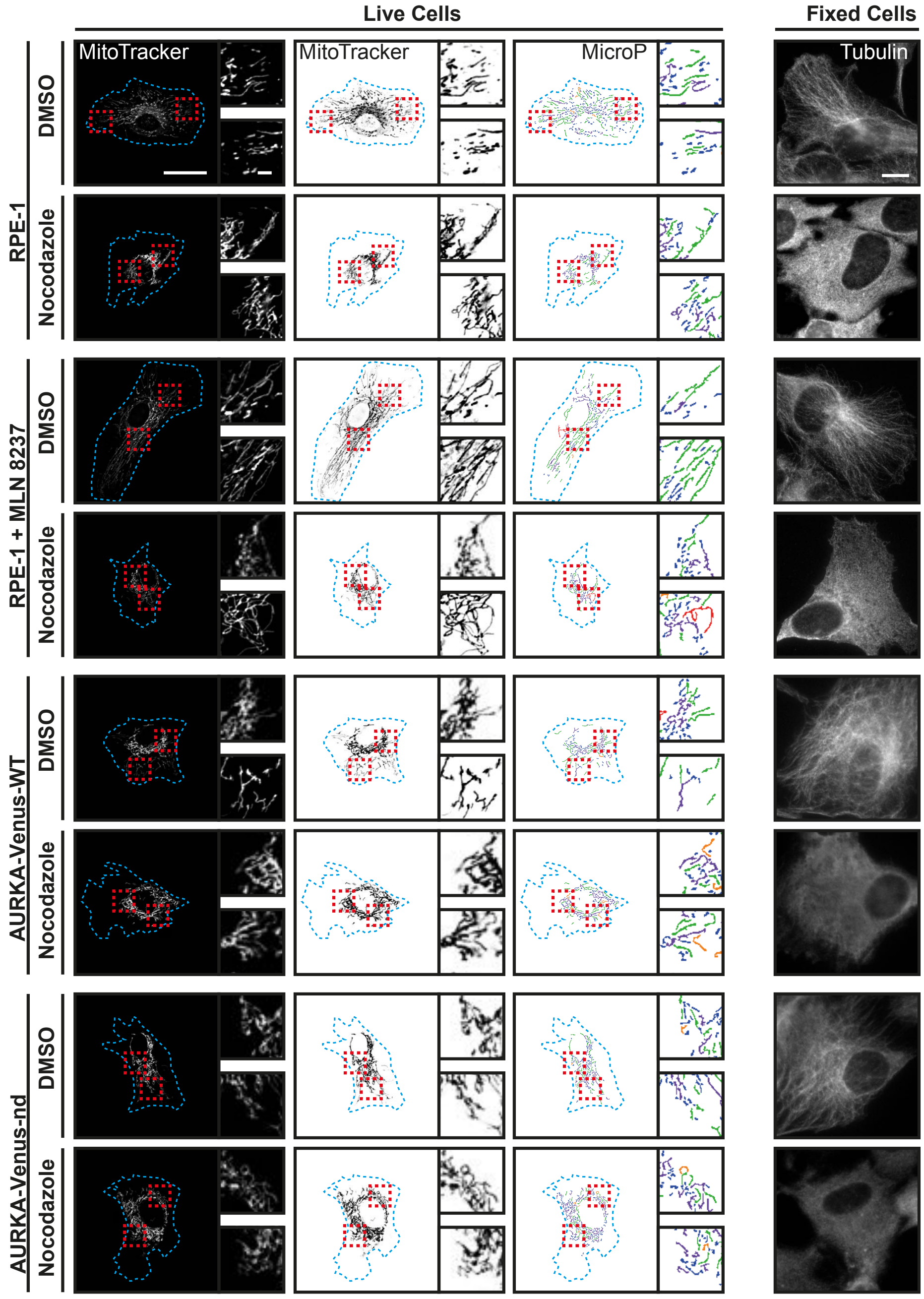

Figure S6: Altered mitochondrial dynamics in response to AURKA are independent of microtubule dynamics. Parental RPE-1 cells (± 100 nM MLN8237 treatment, 3 hours) and cell lines induced for expression of WT- and nd- AURKA-Venus for 24 h were treated for 30 min with 1.5 μM colcemid or 500 ng/ml nocodazole, or for 60 min with 5 μM taxol. Cells were then either stained with MitoTracker® for live imaging of mitochondria (**A-C**) or fixed and stained for microtubules with an antibody against β-tubulin (**B-C**). **A** Scatter plots of individual mitochondrial lengths (30 mitochondria/ cell, n≥ 20 cells per condition) with mean ± S.D. indicated (left hand panel) or as kernel density plots to show size distribution (right hand panel). *p*-values within each set DMSO/colcemid/nocodazole/taxol = n.s.; *p*-value between sets (DMSO treatment in parental/MLN-treated/AURKA-wt/AURKA-nd) < 0.0001 (One-way Anova). **B-C** Illustrative panels

Journal of Cell Science • Supplementary information

of cells treated with taxol (**B**) or nocodazole (**C**). From left to right in each set of panels: Image of MitoTracker-stained cell with magnified inserts, inverted image for enhanced visualization of mitochondria with magnified inserts, MicroP analysis of MitoTracker staining with magnified inserts, immunofluorescence detection of microtubules in a methanol-fixed cell from a population treated in parallel with the same drugs. MicroP colour code for mitochondrial morphology: blue, small globular; green, simple tubular; purple, branching tubular; orange, twisting tubular; red, donuts, loops and non-resolvable segments.

Journal of Cell Science • Supplementary information
