# Supplementary material for: AURKA destruction is decoupled from its activity at mitotic exit but is essential to suppress interphase activity
Source: J Cell Sci. 2020 Jun 16;133(12):jcs243071. doi: 10.1242/jcs.243071 (PMC7328152; doi:10.1242/jcs.243071)
Supplement: Reviewer comments [file joces-133-243071_review_history.pdf]

Journal of Cell Science

AURKA destruction is decoupled from its activity at mitotic exit but is essential to suppress interphase activity

Ahmed Abdelbaki, H. Begum Akman, Marion Poteau, Rhys Grant, Olivier Gavet, Giulia Guarguaglini and Catherine Lindon
DOI: 10.1242/jcs.243071

Editor: David Glover

Review timeline

Original submission:	19 December 2019
Editorial decision:	27 January 2020
First revision received:	5 March 2020
Editorial decision:	6 April 2020
Second revision received:	15 April 2020
Accepted:	19 April 2020

Original submission

First decision letter

MS ID#: JOCES/2019/243071

MS TITLE: AURKA destruction is decoupled from its activity at mitotic exit but essential to suppress interphase activity

AUTHORS: Ahmed Mohamed Abdelbaki Abdelaal, Hesna Begum Akman Tuncer, Marion Poteau, Rhys Grant, Giulia Guarguaglini, Olivier C.G Gavet, and Catherine Lindon
ARTICLE TYPE: Research Article

We have now reached a decision on the above manuscript.

To see the reviewers' reports and a copy of this decision letter, please go to: https://submit-jcs.biologists.org and click on the 'Manuscripts with Decisions' queue in the Author Area. (Corresponding author only has access to reviews.)

As you will see, the reviewers raise a number of substantial criticisms that prevent me from accepting the paper at this stage. They suggest, however, that a revised version might prove acceptable, if you can address their concerns. If you think that you can deal satisfactorily with the criticisms on revision, I would be pleased to see a revised manuscript. We would then return it to the reviewers.

Please ensure that you clearly highlight all changes made in the revised manuscript. Please avoid using 'Tracked changes' in Word files as these are lost in PDF conversion.

I should be grateful if you would also provide a point-by-point response detailing how you have dealt with the points raised by the reviewers in the 'Response to Reviewers' box. Please attend to all of the reviewers' comments. If you do not agree with any of their criticisms or suggestions please explain clearly why this is so.

© 2020. Published by The Company of Biologists under the terms of the Creative Commons Attribution License (https://creativecommons.org/licenses/by/4.0/).

Reviewer 1

Advance summary and potential significance to field

The authors present a solid study that makes a number of interesting observations. The key question at the heart of the story seems to be how exactly is Aurora A suppressed as cells exit mitosis, and in particular what is the relative importance of degradation versus dephosphorylation. But then the rather different concept pops up at the end about how mitochondria are equally segregated during cell division.

The first key result is in Figure 2B where the authors show that in a Cdh1-deficient cell line despite Aurora A protein levels persisting, phosphorylation on T288 diminishes as expected.
This is supported by 2C where the authors show that as cells transition from metaphase to anaphase, T288 phosphorylation on the poles diminishes similarly in parental and Cdh1-deficient cells, even though total Aurora A signal is maintained in the Cdh1-deficient cells.
Next the authors use a FRET-based biosensor as a proxy for Aurora A activity. Data in 3D indicates that late mitotic diminution of Aurora A occurs equally in parental and Cdh1-deficient cells, again suggesting that Cdh1 is not required to suppress Aurora A's kinase activity.

The authors then turn to Tpx2, but the authors really need to explain better why they do what they do next. "Since transient overexpression of full-length TPX2 inhibited mitotic progression in our hands, …", ok, but what are you trying to do here? The question/hypothesis needs spelling out before you then go on to explain that overexpressing Tpx2 or a fragment thereof is the way to test this hypothesis. Nevertheless, a key observation follows. In Cdh1 mutant cells, overexpression of Tpx2 delays T288 dephosphorylation, suggesting that Tpx2 is protecting Aurora A and that the requirement of APC-mediated proteolysis to downregulate Aurora A is via degradation of Tpx2 rather than Aurora A itself.

The authors then turn to mitochondria, but the novelty here is limited; "We and others have previously described a role for AURKA, at physiologically relevant levels of expression, in promoting mitochondrial fission during interphase". The new angle seems to be that inhibiting post-mitotic suppression of Aurora A impacts on mitochondrial morphology. And then, you have to get to the final few lines in the Discussion to discover the interesting aspect of the paper: "Increased rates of fission occur in preparation for mitosis so that mitochondrial fragments can be equally distributed between daughter cells. Here we show that the presence of non-degraded AURKA delays reassembly of the mitochondrial network after cell division.
Whilst there is increasing evidence that the dynamic state of mitochondria contributes to the overall metabolic state of the cell, our understanding of how this impacts progression in the cell cycle remains limited [44, 45]. Our study provides new evidence that APC/C-FZR1 control of AURKA activity in interphase is a critical parameter in regulation of mitochondrial dynamics."

Comments for the author

Overall, the data supports the conclusions, but the manuscript would benefit from considerable polishing to improve the data presentation, language and impact.

The presentation of the graphs should be standardized; it's presently a mix of bars, scatter plots and box-and-whisker plots. For the stats, we seem to be looking at cells within single experiments, are these backed up by biological replicates?

In the Introduction, the authors state that APC-Cdc20 degrades cyclin B and securin, then APC-Cdh1 degrades all the remaining substrates including Aurora kinases. Is this true? For example, while Cenp-F degradation initiates post-anaphase, it is dependent on Cdc20 rather than Cdh1 (Gurden et al, J Cell Sci 2010).

The authors state that "We observed, however, that biosensor activity starts to increase again gradually in G1 in FZR1KO cells compared to parental U2OS cells." However, it looks like FRET signal in 3D increases in both parental and Cdh1 mutants, but that the timing of the switch is different; in parental cells it starts to increase after ~110 mins versus ~65 minutes in Cdh1 mutant cells.

© 2020. Published by The Company of Biologists under the terms of the Creative Commons Attribution License (https://creativecommons.org/licenses/by/4.0/).

The language really needs to be tightened up throughout. Examples include, but are not limited to:
Use of the expression “to look” to describe an IF experiment.
“Using a SAC arrest/release protocol” – say what you did, you used an Mps1 inhibitor to override SAC-mediated mitotic arrest.
“ …to provide a more comprehensive readout of AURKA activity.” The point is to try to measure Aurora A activity in living single cells as they progress through mitosis, versus analysis of a population of synchronised cells fixed at various time points.
Several references to “biosensor activity”; the biosensor itself has no activity, it is a proxy for Aurora A activity.

Bottom of page 3 “We concluded that inactivation of AURKA through dephosphorylation of its T-loop occurs at around the time of onset of AURKA destruction, …” Then top of page 4
“Since the kinetics of dephosphorylation and destruction of AURKA in mitotic exit were not identical (Figure 1E), …” Are these two statements not contradictory?

Reviewer 2

Advance summary and potential significance to field

In the present paper, Lindon and co-workers investigate the link between AURKA degradation and activity during mitotic exit and interphase by means of a new FZR1 KO (CRISPR-Cas9) and a novel FRET-based AURKA biosensor. The rationale is that FZR1 was previously shown to be required for AURKA degradation. The authors now show that AURKA is still inactivated as cells exit mitosis despite not being degraded. This could be explained by Cdc20-dependent degradation of TPX2, a known AURKA activator. To investigate the consequences of not degrading AURKA during interphase, the authors investigate the respective impact on mitochondrial dynamics. They provide convincing evidence that mitochondrial fragmentation during interphase is regulated by AURKA degradation. Overall, I find this a well-thought and executed study that clarifies the regulation of an important cell cycle kinase and would be of interest for publication in Journal of Cell Science. I have only a few specific concerns that I would recommend to be addressed prior to publication.

Comments for the author

Essential reivsions:
1- Fig. 2A: the authors interpret the data as if AURKA levels remain constant in FZR1 KO through mitotic exit. However, high AURKA levels might prevent cells from exiting mitosis. Although changes in cell morphology suggest that cells indeed were exiting mitosis without AURKA degradation (and without FZR1), it is difficult to conclude so in the absence of a DNA marker to unequivocally show that chromosomes are decondensing normally in the absence of FZR1. Incubation of the cells as they exit mitosis with a live DNA dye, such as SiR-DNA, would provide such demonstration.
2- Mitotic exit assay using SAC inhibitor: this is a nice and convenient way to monitor what is going on in a synchronized cell population through mitotic exit. However, because normal mitotic exit (i.e. the interval between anaphase onset and nuclear envelope reassembly/telophase) in human cells takes less than 10 min, I wonder if the effects that the authors are looking in this assay truly reflect mitotic exit or a post-mitotic G1 event. For instance, when we compare time zero with 15 min after Mps1 inhibition, the effects on substrates such as TPX2, AURKA itself, Cdc20, or even AURKA activation is negligible even in control cells. The authors should determine how long does it take for STLC-treated cells to exit mitosis after acute Mps1 inhibition. If cells exit in less than 15 min, a significant part of the data has to be re-interpreted, and Aurora A inactivation might only really take place during interphase.

Minor issues:

1- Introduction: bottom of page 2, the authors suggest that cyclin B is fully degraded by APC/C-CDC20 during metaphase. However, recent work by Afonso et al., eLife, 2019 suggests that cyclin B continues to be degraded during anaphase, at least in part mediated by APC/C-Cdh1. This should be corrected.

© 2020. Published by The Company of Biologists under the terms of the Creative Commons Attribution License (https://creativecommons.org/licenses/by/4.0/).

2- Were the results consistent among different FZR1 KO clones?

First revision

Author response to reviewers' comments

Response to reviewers:

We were pleased that both reviewers found our story interesting, and that our data supported the conclusions drawn. We thank them for their critique and for some very useful comments, and we hope they will agree that the revisions we have carried out in response have improved the manuscript.
We outline the changes we have made in our point by point response to the reviewers below:

Reviewer 1
We took on board the reviewer's general comments

- that the reader is not well prepared for the final part of the story, about mitochondrial regulation.

We have added (1) a sentence to the Introduction that states clearly that the regulation of mitochondrial homeostasis is an important function of AURKA relevant to the question of how its activity is regulated in interphase. (2) a sentence in the Discussion that highlights reassembly of the mitochondrial network as a key step in post-mitotic reconstitution of the interphase state.

- that the TPX2 overexpression experiment was not properly explained.

We have now added a sentence to this section to state a clear hypothesis.

In response to specific comments:

Reviewer 1 Comments for the Author:
1) Overall, the data supports the conclusions, but the manuscript would benefit from considerable polishing to improve the data presentation, language and impact.
We have extensively reviewed the presentation of data (see point 2) and language and impact (see point 5) throughout the manuscript

2) The presentation of the graphs should be standardized; it's presently a mix of bars, scatter plots and box-and-whisker plots. For the stats, we seem to be looking at cells within single experiments, are these backed up by biological replicates?
We have replaced several graphs with scatter plots - Figures 5A, 6B, S3B (now S4B), S4A (now S5A), S4B (now S5B), a new plot S5F, and S5A (now S6A).
Scatter plots in Figures 1 and 2 contain pooled data, but otherwise graphs generally contain data from single experiments, with these representative of two or more biological replicates (details in figure legends). For the IF quantifications shown in Figure 1, we pooled data from two experiments because there were few data points from anaphase cells, and since these gave highly variable staining we wanted to show as much of the variability as possible. The data points were pooled from 2 experiments, and another 2 experiments were carried out with similar results. The plot in Figure 2E also shows the same result. For mitochondrial length quantifications, scatter plots report on mean values for 30 measurements per cell, as described in the Materials and Methods, and are representative of two or more biological replicates.

3) In the Introduction, the authors state that APC-Cdc20 degrades cyclin B and securin then APC-Cdh1 degrades all the remaining substrates, including Aurora kinases. Is this true? For example, while Cenp-F degradation initiates post-anaphase, it is dependent on Cdc20 rather than Cdh1 (Gurden et al, J Cell Sci 2010).
We agree with the reviewer on this point, and certainly did not intend the interpretation taken. Since our phrase 'switch in specificity of Cdc20' has been understood as a switch from Cdc20 to Cdh1, we have altered the text (to remove the word 'switch') at bottom of page 2, and hope this is now clearer.

© 2020. Published by The Company of Biologists under the terms of the Creative Commons Attribution License (https://creativecommons.org/licenses/by/4.0/).

4) The authors state that "We observed, however, that biosensor activity starts to increase again gradually in G1 in FZR1KO cells compared to parental U2OS cells." However, it looks like FRET signal in 3D increases in both parental and Cdh1 mutants, but that the timing of the switch is different; in parental cells it starts to increase after ~110 mins versus ~65 minutes in Cdh1 mutant cells.

Yes, this is a better description of the results and we have modified the text accordingly. Bottom of p5 now reads "*We observed, however, that FZR1^{KO} cells built up AURKA activity after mitosis earlier than parental cells and reached a significantly higher level in G1 phase (Figure 3D). We concluded that destruction of AURKA does not influence its inactivation during mitotic exit but may be important to prevent premature re-activation early in the cell cycle.*"

5) The language really needs to be tightened up throughout. Examples include but are not limited to:

Use of the expression "to look" to describe an IF experiment.

Now replaced.

"Using a SAC arrest/release protocol" - say what you did, you used and Mps1 inhibitor to override SAC-mediated mitotic arrest.

Now changed as suggested (top of p4).

" ...to provide a more comprehensive readout of AURKA activity." The point is to try to measure Aurora A activity in living single cells as they progress through mitosis, versus analysis of a population of synchronised cells fixed at various time points.

By 'comprehensive' we were trying to say 'cell-wide' (in contrast to localized biosensors that have been used in other studies). We have now modified text at top of p5 to explain this better: *"toprovide a cell-wide readout of AURKA activity in living single cells as they progress through mitosis"*

Several references to "biosensor activity"; the biosensor itself has no activity, it is a proxy for Aurora A activity.

Here we were using 'activity' as shorthand for 'FRET activity', but we agree this was sloppy language and have changed it wherever it occurred (with FRET measurement, FRET signal or 1/FRET signal, as appropriate)

Bottom of page 3 "We concluded that inactivation of AURKA through dephosphorylation of its T-loop occurs at around the time of onset of AURKA destruction, ..." Then top of page 4 "Since the kinetics of dephosphorylation and destruction of AURKA in mitotic exit were not identical (Figure 1E), ..." Are these two statements not contradictory?

We had hoped that 'around the time' was ambiguous enough to avoid contradiction, but we have modified the text from bottom of p3 to be clearer, as follow:

1) "We concluded that inactivation of AURKA through dephosphorylation of its T-loop occurs at approximately the time of onset of AURKA destruction, which occurs around 10 minutes after anaphase onset in human cells [28]. We further tested our conclusion by immunoblot analysis of extracts ....

2) ....confirmed that pT288 levels and total AURKA both fall rapidly during forced mitotic exit (Figure 1E), although at different rates.

We also extensively revised language around presentation of figure data and interpretation throughout the Results section.

Reviewer 2

Essential revisions:

1- Fig. 2A: the authors interpret the data as if AURKA levels remain constant in FZR1 KO through mitotic exit. However, high AURKA levels might prevent cells from exiting mitosis. Although changes in cell morphology suggest that cells indeed were exiting mitosis without AURKA degradation (and without FZR1), it is difficult to conclude so in the absence of a DNA marker to unequivocally show that chromosomes are decondensing normally in the absence of FZR1. Incubation of the cells as they exit mitosis with a live DNA dye, such as SiR-DNA, would provide such demonstration.

We had timelapse movies from parental and FZR1^{KO} cells expressing H2B-GFP showing that mitotic exit timings look the same in these cell lines, and have now scored these movies and

© 2020. Published by The Company of Biologists under the terms of the Creative Commons Attribution License (https://creativecommons.org/licenses/by/4.0/).

added the data to Figure 2 (new Figure 2B).
We carried out a new experiment using SiR-DNA dye to film cells undergoing forced mitotic exit (in response to reviewer 2's point (2) below) and this also showed no difference in the timing of DNA decondensation in absence of FZR1.
We would also point out that use of cyclin B1 as a marker of mitotic exit in immunoblots (previously Figure 2B, Figure 2C in the revised version) shows that there is no delay in mitotic exit due to FZR1^{KO}.

2- Mitotic exit assay using SAC inhibitor: this is a nice and convenient way to monitor what is going on in a synchronized cell population through mitotic exit. However, because normal mitotic exit (i.e. the interval between anaphase onset and nuclear envelope reassembly/telophase) in human cells takes less than 10 min, I wonder if the effects that the authors are looking in this assay truly reflect mitotic exit or a post-mitotic G1 event. For instance, when we compare time zero with 15 min after Mps1 inhibition, the effects on substrates such as TPX2, AURKA itself, Cdc20, or even AURKA activation is negligible even in control cells. The authors should determine how long does it take for STLC-treated cells to exit mitosis after acute Mps1 inhibition. If cells exit in less than 15 min, a significant part of the data has to be re-interpreted and Aurora A inactivation might only really take place during interphase.
This is an interesting point for discussion that we have debated amongst ourselves.
We think there is enough data elsewhere in the paper (e.g. the IF data in Figure 1C,D) to conclude that AURKA inactivation is a mitotic exit event (i.e. that it is already occurring as cells transition telophase, within 10-15 minutes of anaphase onset). The use of cyclin B1 as a readout for mitotic exit in forced mitotic exit (immunoblots in Figure 2C of revised version) confirms that under these conditions anaphase onset should be within 15 mins of Mps1 addition. We carried out a new timelapse experiment of cells undergoing forced mitotic exit in the presence of SiR-DNA dye in order to visualise the chromatin and found that the time taken for cells to enter 'C-phase' (the period of cortical contractility that accompanies anaphase onset) was approximately 15 minutes, consistent with the timing of cyclin B1 disappearance we see by immunoblot. The time taken to complete forced mitotic exit (as scored by either cell respreading, or by chromatin decondensation/NE reassembly) was about 90 minutes, which is longer than the AURKA inactivation window defined by loss of pT288 in immunoblot.
We agree that the time between anaphase and telophase in human cells is normally less than 10 minutes, but would argue that this is not the 'end' of mitotic exit. The time taken for the cell to transition to full interphase state (reversal of all mitotic cdk phosphorylation events, full decondensation of chromatin etc) is longer, even in an unperturbed mitosis. In our forced mitotic exit protocol there is additionally some lack of synchrony (i.e. variability in timing of the SAC override) that also makes the apparent timing of mitotic exit, and associated events, look slower.

We have modified the text at top of p4 to acknowledge that we don't know exactly when the AURKA inactivation window closes: *"pT288 levels and total AURKA both fall rapidly during forced mitotic exit"* changed to *"pT288 levels and total AURKA both fall rapidly <u>after</u> forced mitotic exit"*.

Minor issues:
1- Introduction: bottom of page 2, the authors suggest that cyclin B is fully degraded by APC/C- CDC20 during metaphase. However, recent work by Afonso et al., eLife, 2019 suggest that cyclin B continues to be degraded during anaphase, at least in part mediated by APC/C-Cdh1. This should be corrected.
In fact we said in the original version "cyclin B1 is targeted to achieve anaphase onset" and did not mean to suggest it is fully degraded, only that it is cyclin B1 degradation that allows anaphase onset. However we have removed the possibility of this interpretation by spelling out in more detail the regulation of APC/C at anaphase and citing the suggested reference. This section now reads: *"APC/C-CDC20 initially targets cyclin B and securin during metaphase to drive chromosome segregation and mitotic exit. After anaphase onset altered substrate specificity of APC/C-CDC20 and activation of APC/C- FZR1 together control degradation of the remaining pool of cyclin B (Afonso 2019) as well as other APC/C substrates including Aurora kinases (Lindon 2015)"*

© 2020. Published by The Company of Biologists under the terms of the Creative Commons Attribution License (https://creativecommons.org/licenses/by/4.0/).

2- Were the results consistent among different FZR1 KO clones?
We have immunoblots from a second clone to show that the relative dynamics of pT288 versus AURKA during mitotic exit are the same in this clone as in Figure 2C, and have added these blots to Figure S2. We carried out a new experiment to test mitochondrial fragmentation in this clone. We found that fragmentation was even more pronounced than in the original clone, and also rescued by treatment with MLN8237. We have added this new data to Figure S5.

Second decision letter

MS ID#: JOCES/2019/243071

MS TITLE: AURKA destruction is decoupled from its activity at mitotic exit but essential to suppress interphase activity

AUTHORS: Ahmed Mohamed Abdelbaki Abdelaal, Hesna Begum Akman Tuncer, Marion Poteau, Rhys Grant, GIULIA GUARGUAGLINI, Olivier C.G Gavet, and Catherine Lindon
ARTICLE TYPE: Research Article

We have now reached a decision on the above manuscript.

To see the reviewers' reports and a copy of this decision letter, please go to: https://submit-jcs.biologists.org and click on the 'Manuscripts with Decisions' queue in the Author Area. (Corresponding author only has access to reviews.)

As you will see, the reviewers gave favourable reports but raised some critical points that will require amendments to your manuscript. I hope that you will be able to carry these out, because I would like to be able to accept your paper.

We are aware that you may currently be unable to access the lab to undertake experimental revisions. If it would be helpful, we encourage you to contact us to discuss your revision in greater detail. Please send us a point-by-point response indicating where you are able to address concerns raised (either experimentally or by changes to the text) and where you will not be able to do so within the normal timeframe of a revision. We will then provide further guidance. Please also note that we are happy to extend revision timeframes as necessary.

Please ensure that you clearly highlight all changes made in the revised manuscript. Please avoid using 'Tracked changes' in Word files as these are lost in PDF conversion.

I should be grateful if you would also provide a point-by-point response detailing how you have dealt with the points raised by the reviewers in the 'Response to Reviewers' box. Please attend to all of the reviewers' comments. If you do not agree with any of their criticisms or suggestions please explain clearly why this is so.

Reviewer 1

Advance summary and potential significance to field

This is a revised version of a manuscript focusing on the mechanisms of Aurora A degradation.

Comments for the author

While the first version was solid I felt that the presentation could be improved. This the authors have done. They introduce earlier the link to mitochondria, the language and interpretation has been tightened up considerably.

© 2020. Published by The Company of Biologists under the terms of the Creative Commons Attribution License (https://creativecommons.org/licenses/by/4.0/).

In addition, it is now more clear in the legends how many biological replicates were performed etc. Consequently it is now suitable for publication in J. Cell Sci.

Reviewer 2

Advance summary and potential significance to field

The authors did a good job addressing my previous concerns and I can now recommend publication in JCS pending the address of the minor issue below.

Comments for the author

I still think that taking 90 min for mitotic exit is an overestimation for these cells. By definition, the reassembly of the nuclear envelope determines the compartimentalization of the nucleus and the end of mitosis, and this does not take more than 15 min in human cells. Sure, the cytoplasm continues to undergo changes to re-establish adhesion to the substrate and complete cytokinesis. Sure, DNA continues to decondense and the microtubule cytoskeleton continues to re-organize. I think it is important to mention exactly how the authors determined/considered mitotic exit and explicitly state in the manuscript that the events that are likely regulated by Aurora A significantly extend beyond the normal duration of anaphase and the time of re-assembly of the nuclear envelope, which defines telophase. Thus, they are post-telophase events. I note this to avoid confusion because for most people, anaphase is considered mitotic exit.

Second revision

Author response to reviewers' comments

Dear David,

Many thanks to you and the reviewers of our manuscript "**AURKA destruction is decoupled from its activity at mitotic exit but essential to suppress interphase activity**" for completing their assessment under difficult conditions. We are pleased that both reviewers were satisfied with the revised version. We have made a small number of additional modifications to the text in response to the remaining concern of reviewer 2, that our definition of mitotic exit was unclear. In reply to reviewer 2's comment:

1) IF experiments (Fig. 1C,D) appear to show that most AURKA inactivation occurs by the end of telophase, consistent with the traditional definition of mitotic exit (and within 20 minutes of anaphase onset).

2) Under 'forced' mitotic exit conditions, pT288 disappearance by immunoblot (e.g. Figs 1E, 2C) appears to have slower timing (a) because anaphase onset does not occur until 15-20 minutes following Mps1 inhibition (we have now made this clearer to readers by drawing attention to the timing of cyclin B1 degradation) and (b) because some lack of synchrony in mitotic exit also 'spreads the peak', contributing to making events look slower than they do in single cell assays.

3) Use of the FRET-based biosensor also indicates slower inactivation kinetics than that we see by IF with pT288 antibody, but the biosensor is measuring AURKA activity independent of pT288.

4) Since the exact timing of events varies according to the assay used to study it, we have deliberately used the general term 'mitotic exit' to describe this window of time. Although the reviewer considers mitotic exit complete once the nuclear envelope is re-assembled, we prefer more physiological definitions of mitosis that would consider full reversal of mitotic CDK substrate phosphorylation as the event marking transition from mitosis to interphase.

© 2020. Published by The Company of Biologists under the terms of the Creative Commons Attribution License (https://creativecommons.org/licenses/by/4.0/).

5) However, In order to avoid any confusion for the reader we have made modifications to the manuscript to acknowledge that AURKA inactivation may be a process that continues beyond the end of mitosis, irrespective of definition:

• we altered the Abstract to read "Activity peaks at mitosis before AURKA is degraded during ***and after*** mitotic exit in a process strictly dependent on APC/C coactivator FZR1"
• we modified the description of Figure 1E in the results section to read: "Immunoblotting of cell extracts shows that under these conditions of 'forced' mitotic exit, ***cells significantly degrade cyclin B1 - the trigger for anaphase entry - within 15 minutes (Figure 1E). The fall in pT288 and total AURKA levels are delayed relative to cyclin B1 degradation, consistent with events ongoing through anaphase and telophase into following G1 phase***.
• we modified the first paragraph of the Discussion section to read: "AURKA activity increases in preparation for mitosis in parallel with the protein level, and both progressively drop from anaphase onwards. This has led to the expectation that destruction contributes to regulating AURKA activity at mitotic exit (Afonso et al., 2017; Floyd et al., 2008). In this study, however, we found that loss of AURKA activity and destruction of the protein are uncoupled. Lack of the APC/C co-activator FZR1 completely stabilizes AURKA levels but ***does not affect the timing of AURKA inactivation measured during mitotic exit and into subsequent G1 phase***, using pT288 reactivity or a novel FRET biosensor for AURKA activity that we characterise in this study. Therefore, AURKA destruction is not required for timing of its inactivation ***after*** mitosis."

We trust that our work can now be considered suitable for publication in J. Cell Science.

With very best wishes,
Cath Lindon

Third decision letter

MS ID#: JOCES/2019/243071

MS TITLE: AURKA destruction is decoupled from its activity at mitotic exit but essential to suppress interphase activity

AUTHORS: Ahmed Mohamed Abdelbaki Abdelaal, Hesna Begum Akman Tuncer, Marion Poteau, Rhys Grant, GIULIA GUARGUAGLINI, Olivier C.G Gavet, and Catherine Lindon
ARTICLE TYPE: Research Article

I am happy to tell you that your manuscript has been accepted for publication in Journal of Cell Science, pending standard ethics checks.

© 2020. Published by The Company of Biologists under the terms of the Creative Commons Attribution License (https://creativecommons.org/licenses/by/4.0/).
